# Racial Disparities in Outcomes of Delivery and Cardiac Complications Among Pregnant Women with Congenital Heart Disease

**DOI:** 10.1007/s40615-024-01950-0

**Published:** 2024-02-28

**Authors:** John Petersen, Waiel Abusnina, Sandeep Beesabathina, Sai Subhakar Desu, Ryan W. Walters, Venkata Mahesh Alla

**Affiliations:** 1 Creighton University School of Medicine, Omaha, NE USA; 2 Section of Interventional Cardiology, MedStar Washington Hospital Center, Washington, DC USA; 3 Saint Louis University School of Medicine, St. Louis, MO USA; 4 Sri Siddhartha Medical College, Tumkur, Karnataka India; 5 Department of Clinical Research and Public Health, Creighton University School of Medicine, Omaha, NE USA; 6 Division of Cardiology, Creighton University School of Medicine, 7710 Mercy Rd., Suite #401, Omaha, NE 68123 USA

**Keywords:** Racial disparities, Pregnancy, Congenital heart disease, Cardiovascular outcomes

## Abstract

Advances in cardiology have led to improved survival among patients with congenital heart disease (CHD). Racial disparities in cardiovascular and maternal outcomes are well known and are likely to be more profound among pregnant women with CHD. Using the 2001 to 2018 National Inpatient Sample, we identified all hospitalizations for delivery among women ≥ 18 years of age with CHD. Unadjusted and adjusted between-race differences in adverse maternal cardiovascular, obstetric, and fetal events were assessed using logistic regression models. During the study period, we identified 52,711 hospitalizations for delivery among women with concomitant CHD. Of these, 66%, 11%, and 16% were White, Black, and Hispanic, respectively. Obstetric complications and fetal adverse events were higher among Blacks compared to Whites and Hispanics (44% vs. 33% vs. 37%, *p* < .001; 36% vs. 28% vs. 30%, *p* < .001), respectively. No between-race differences were observed in overall cardiovascular adverse events (27% vs. 24% vs. 23%, *p* < .21). However, heart failure was significantly higher among Black women (3.6% vs. 1.7% vs. 2.2%, *p* = 0.001). While a lower income quartile was associated with higher rates of adverse outcomes, adjustment for income did not attenuate the adverse impact of race. Black females with CHD diagnoses were more likely to experience adverse obstetric, fetal events, and heart failure compared to White and Hispanic women irrespective of their income status. Further research is needed to identify causes and devise interventions to mitigate racial disparities in the care of pregnant women with CHD.

**Supplementary Information:**

The online version contains supplementary material available at 10.1007/s40615-024-01950-0.

## Introduction

It is estimated that there are around 1.4 million adults living with congenital heart disease (CHD) in the USA [1]. Advances in surgery and care of these patients have resulted in significant improvement in survival, and it is estimated that 95% of babies born with non-critical CHD and 70% born with critical CHD survive to 18 years of age [2]. The number of pregnancies in women with CHD has therefore been increasing in the last few decades and this trend is expected to continue [3]. Inadequate adaption to the hemodynamic stress of pregnancy significantly increases the risk for adverse obstetric, maternal, and fetal outcomes in pregnant women with CHD [4–6]. Appropriate prenatal, intranatal, and postnatal care from a multi-disciplinary pregnancy-heart team consisting of obstetricians, cardiologists, and anesthesiologists with experience in caring for mothers with CHD is critical for optimal maternal and fetal outcomes and is endorsed by multiple professional guidelines [7, 8]. Racial, ethnic, and economic disparities in health care access, care, and outcomes have been well documented in a number of cardiovascular disorders [9–13], and similar disparities in the care and outcomes of pregnancy are also well established [14–19]. Given the known higher risk of adverse outcomes in pregnant women with CHD, the impact of racial and socioeconomic inequities is likely to be more profound. Therefore, in this study, we explored the association of race and socioeconomic status on the cardiovascular, maternal, and fetal outcomes of pregnant women with CHD using a large nationally representative database.

## Methods

All hospitalizations were abstracted from the 2001 to 2018 National (Nationwide) Inpatient Sample (NIS). The NIS is the largest publicly available all-payer inpatient care database in the USA and is part of the Healthcare Cost and Utilization Project (HCUP) family of databases sponsored by the Agency for Healthcare Research and Quality (AHRQ) [20]. When weighted, the NIS contains data on primary and secondary discharge diagnoses and procedures from more than 7 million inpatient hospitalizations annually [20]. This study was acknowledged as Not Human Subjects Research by the Institutional Review Board at Creighton University (InfoEd record number: 2002080).

We identified hospitalizations for childbirth in which the female patient was at least 18 years of age. Hospitalizations for vaginal or cesarean delivery were identified using enhanced childbirth identification methods described previously [4, 21]. Specifically, we used Medicare Severity-Diagnosis Related Groups (MS-DRG) 765, 766, 767, 768, 774, 775; and All Patients Refined-Diagnosis Related Groups (APR-DRG) 540, 541, 542, 560, ICD-9-CM codes V27.x, 650, 669.7x, ICD-10-CM codes Z37.xx, O80, O82, as well as ICD-9-PCS codes 72.xx to 74.xx and ICD-10-PCS codes 10D0- and 10E0-. We excluded any hospitalization resulting from termination of pregnancy (ICD-9-PCS: 74.91; ICD-10-PCS: 10A0-). We identified CHD using relevant ICD-9-CM codes 745.xx to 747.x and ICD-10-CM codes Q20.x to Q28.x. CHD diagnoses were stratified into severe (e.g., Tetralogy of Fallot, hypoplastic heart, and transposition); shunt only (e.g., atrial or ventricular septal defects and patent ductus arteriosus); valve only (e.g., pulmonic, aortic or mitral stenosis); shunt/valve; or other (see Supplemental Table S1 for all ICD-9/10 diagnosis and procedure codes used in this study).

Our outcomes included race-specific differences in cardiovascular events (myocardial infarction, arrhythmia, heart failure, stroke, pulmonary embolism); obstetric events (pregnancy-related hypertension, preeclampsia, eclampsia, preterm delivery, hemorrhage, placental abruption, placenta previa, prolonged pregnancy); and fetal events (malformation, distress, death/stillbirth, growth restriction). In addition, we explored whether socioeconomic status moderated between-race differences by evaluating the two-way interaction between race and median income level. Other outcomes assessed included in-hospital death and length of stay.

For each hospitalization, we extracted patient race (White, Black, Hispanic, other); age, primary payer (medicaid, private, other); income quartile; facility location/teaching status (rural, urban nonteaching, urban teaching); facility bed size (small, medium, large); and the region of the USA in which the facility was located (northeast, midwest, south, west) as well as comorbid conditions that included non-pregnancy related hypertension, heart failure, pulmonary circulation disorder, coronary arterial disease, conduction disorder, diabetes, hyperlipidemia, chronic pulmonary disease, central nervous system disease, obesity, and mental health diagnosis. Further, we calculated the Elixhauser Comorbidity Index from the 29 Elixhauser comorbidities that range from − 32 to 99, in which negative values imply a protective effect and higher positive values imply a harmful effect. The Elixhauser Comorbidity Index is a method of categorizing comorbidities of patients based on the International Classification of Diseases (ICD) diagnosis codes found in administrative data. It is derived based on several comorbid conditions such as congestive heart failure (CHF), hypertension, valvular heart disease, diabetes, obesity, cancer, pulmonary, and renal disorders. categorized dichotomously as either present or absent and has been extensively validated [22, 23].

All descriptive statistics were stratified by race with continuous variables presented as median and interquartile range, and compared using the lognormal regression model. Categorical variables are presented as percent and compared using the Rao-Scott chi-square test; Wilson confidence intervals are presented as appropriate. Unadjusted and adjusted between-race differences in adverse event rates were compared using a logistic regression model; the multivariable models included age, primary payer, income quartile, Elixhauser Comorbidity Index, CHD stratification, congestive heart failure, hypertension, pulmonary circulation disorder, and diabetes. Lognormal regression models were estimated for length of stay given skewed and heteroscedastic residuals. The functional form of continuous covariates was evaluated using restricted cubic splines with knots prespecified at the 5th, 27.5th, 50th, 72.5th, and 95th percentiles; nonlinear effects were retained as dictated by the likelihood ratio test. All analyses were conducted using SAS v. 9.4 and accounted for the NIS sampling design.

## Results

**Fig. 1:**
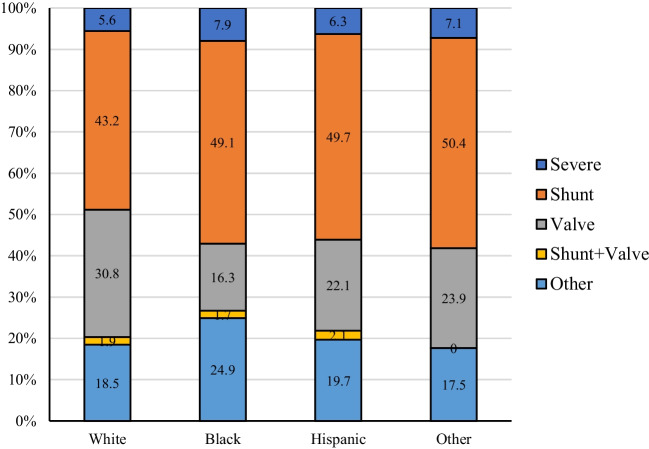
CHD type (as % of total) stratified by race. The percent of Shunt + Valve for hospitalizations in which the patient was of Other race was very low and could not be reported per the NIS Data Use Agreement

From 2001 to 2018, an estimated 69.2 million hospitalizations for vaginal/cesarean delivery occurred in the USA (95% CI, 67.1 million to 71.3 million). An estimated 52,711 of these hospitalizations included a patient with a CHD diagnosis (95% CI, 50,445 to 54,977) with shunt-only lesions (atrial or ventricular septal defects and patent ductus arteriosus) being the most common CHD diagnosis (Fig. 1). The rate of cesarean section was higher with a CHD diagnosis compared to without a CHD diagnosis (37.7% vs. 31.4%, *p* < 0.001).

For delivery-related hospitalizations that included a patient with a CHD diagnosis, an estimated 65.7% (95% CI, 64.3 to 67.0%) were White patients; 10.5% (95% CI, 9.8 to 11.3%) were Black patients; 15.8% (95% CI, 14.7 to 17.0%) were Hispanic patients; and 8.0% (95% CI, 7.4 to 8.7%) included patients of another race. The estimated rate of cesarean section was statistically similar across races (38.2%, 37.1%, 36.8%, and 36.4% for Whites, Blacks, Hispanics, and other races, respectively; *p* = 0.625). Further, an estimated 70.9% of Blacks were below the median income quartile compared to 60.3% in Hispanics, 43.1% in Whites, and 38.6% in other races (Table 1) with Black and Hispanic patients tending to be younger, with higher rates of Medicaid, and more often treated in urban teaching hospitals (Table 1). White patients had lower rates of background systemic and pulmonary hypertension, heart failure, diabetes, obesity, respiratory disorders and more often received care in rural or urban non-teaching hospitals. Whites and Blacks had higher rates of mental health and neurologic disorders compared to Hispanic and other races (Table 2).

**Table 1:** Demographic/hospital characteristics among CHD hospitalizatations for delivery stratified by race

	White	Black	Hispanic	Other	*p*
Age (yrs.) median [IQR]	28 [24–32]	26 [21–31]	27 [22–31]	29 [25–33]	< .001
18–19	5.3	10.3	8.9	5.3	< .001
20–24	21.7	30.3	27.4	16.2	
25–29	29.4	25.9	27.4	26.6	
30–34	27.6	19.8	22.5	32.2	
35–39	12.9	9.9	10.7	14.8	
40 +	3.1	3.8	3.2	4.8	
Insurance					
Medicaid	28.8	62.0	63.6	38.0	< .001
Private	64.5	29.9	29.2	54.4	
Other	6.7	8.1	7.1	7.6	
Income quartile					
I	18.8	48.8	33.0	20.2	< .001
II	24.3	22.1	27.3	18.4	
III	27.2	18.1	23.4	26.2	
IV	29.7	11.0	16.3	35.3	
Location-teaching status					
Rural	8.5	2.5	2.5	5.4	< .001
Urban nonteaching	24.7	13.5	22.3	19.1	
Urban teaching	66.8	83.9	75.2	75.5	
Bed size					
Small	11.7	6.8	9.6	11.3	0.002
Medium	22.9	20.6	20.3	21.0	
Large	65.4	72.6	70.1	67.7	
Region					
Northeast	24.0	19.3	17.7	23.1	< .001
Midwest	22.6	21.6	5.4	13.8	
South	33.8	48.1	32.6	25.2	
West	19.6	11.0	44.3	37.8	

Note: Data presented as %. *CHD* congenital heart disease, *IQR* interquartile range

**Table 2:** Baseline clinical characteristics of patients with CHD admitted for delivery stratified by race

	White	Black	Hispanic	Other	*p*
Elixhauser Comorbidity Index	− 1 [− 1, 0]	− 1 [− 2, 0]	− 1 [− 1, 0]	− 1 [− 1, 0]	< .001
Hypertension	4.8	10.8	5.8	5.5	< .001
Heart failure	1.7	3.9	2.0	2.4	< .001
Pulmonary hypertension	2.1	5.7	6.9	6.6	< .001
CAD	1.1	1.9	1.0	*	-
Conduction/rhythm disorder	8.9	11.1	8.7	8.7	0.142
Other cardiovascular	38.2	27.3	29.6	32.0	< .001
Diabetes	1.4	4.3	2.8	2.6	< .001
Hyperlipidemia	0.6	*	0.8	*	-
Obesity	6.6	11.4	9.7	6.0	< .001
Morbid obesity	3.1	6.2	4.0	2.3	< .001
Mental health	19.0	20.3	10.2	10.2	< .001
Neurologic disorder	14.6	16.3	8.6	7.9	< .001
Respiratory/pulmonary	13.8	22.7	18.4	15.9	< .001

Note: Data presented as count, count (%), %, or median [IQR]. An * indicates that the number of observed hospitalizations was 10 or less, which cannot be presented per the NIS Data Use Agreement. *CHD* congenital heart disease, *CAD* coronary artery disease

**Fig. 2:**
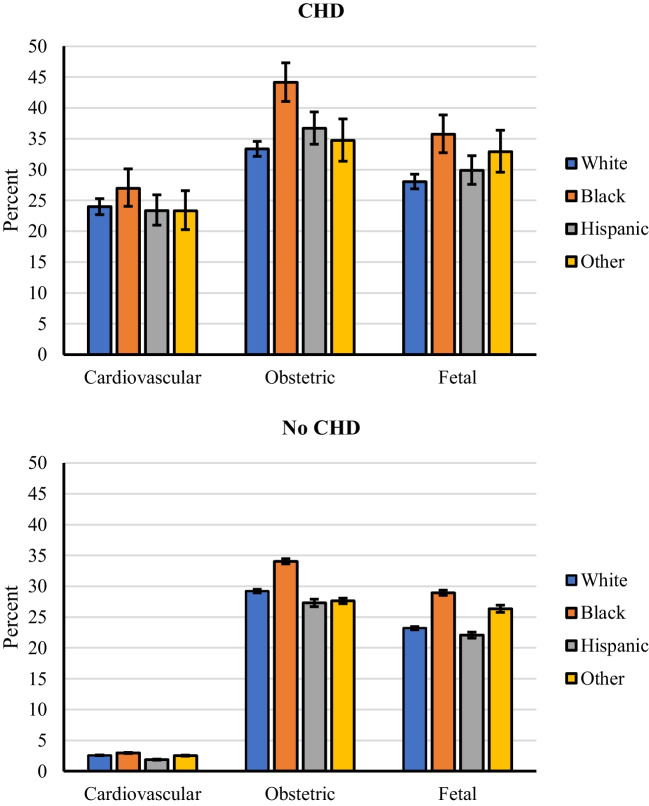
Unadjusted rate of adverse cardiovascular, obstetric, and fetal events stratified by race for hospitalizations in which the patient had CHD (top) or did not have CHD (bottom). Error bars represent 95% confidence intervals

**Fig. 3:**
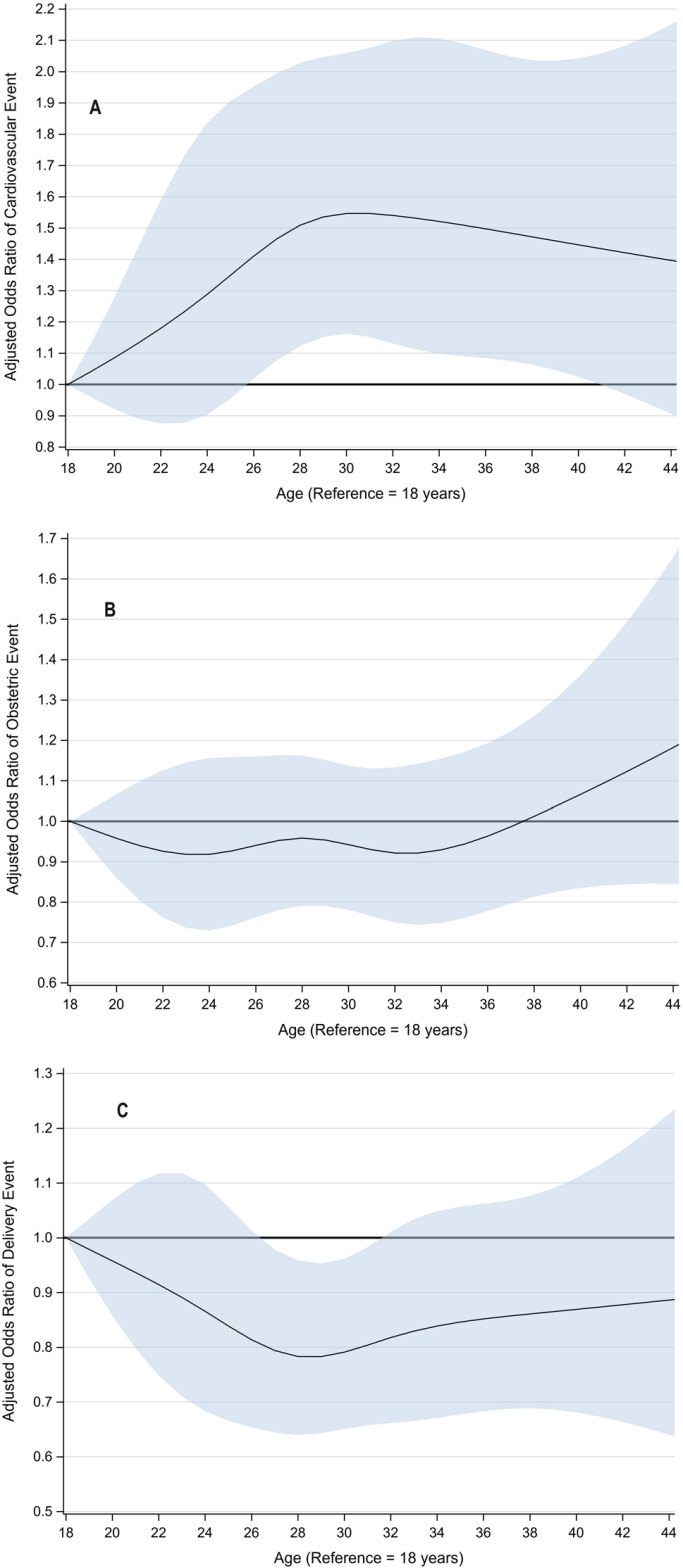
Adjusted odds ratios (thick blue line) by age for adverse cardiovascular (**A**), obstetric (**B**), and fetal (**C**) events. All odds ratios are relative to a reference age of 18 years. Shaded areas represent 95% confidence intervals

**Fig. 4:**
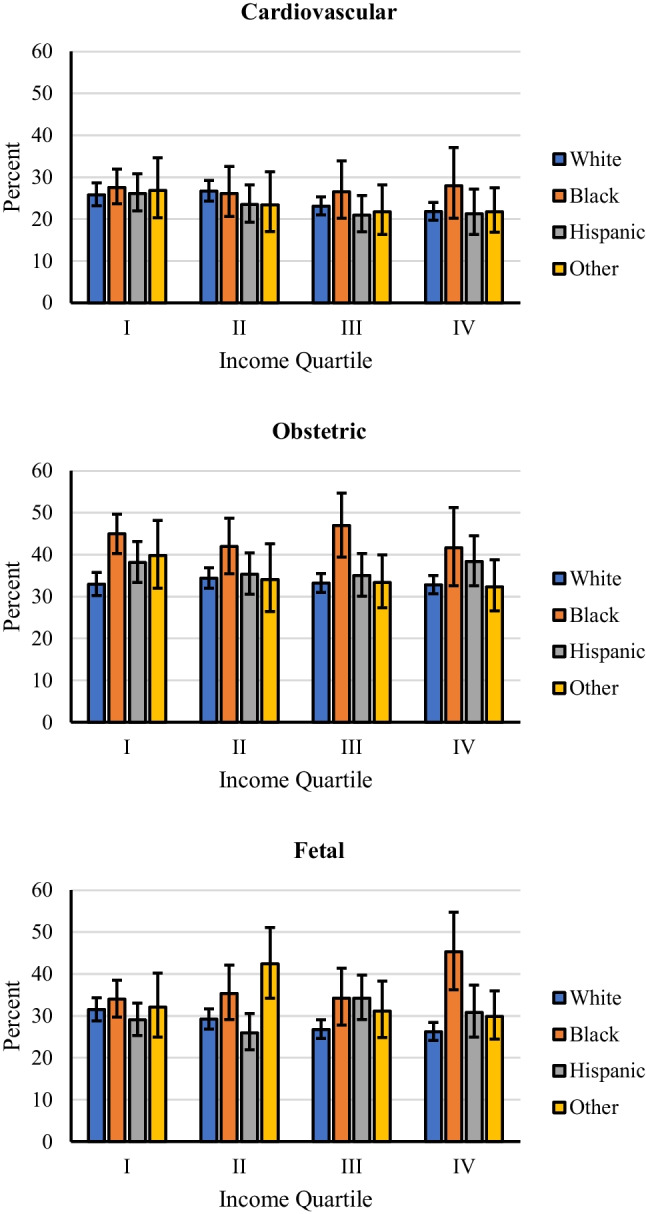
Unadjusted rate of adverse cardiovascular (top), obstetric (middle), and fetal (bottom) events stratified by race and income quartile. Error bars represent 95% confidence intervals

For all races, the unadjusted rates of cardiovascular, obstetric, or fetal adverse events were significantly higher for hospitalizations with CHD diagnosis (all *p* < 0.001; Fig. 2 and Table 3). Importantly, the pattern of between-race differences in adverse events was similar in hospitalizations with or without CHD with Black patients having the highest rates of adverse events irrespective of CHD. An estimated 24.1% (95% CI, 23.1 to 25.3%) of CHD patients had an adverse cardiovascular event. The most common cardiovascular events were thromboembolism followed by arrhythmias and CHF (Table 3). Black patients had a higher rate of adverse cardiovascular events (27.0%) compared to all other races, though this was not statistically significant (24.0% for Whites and 23.3% for Hispanics and another race, respectively, *p* = 0.207; Table 3 and Fig. 2). Between-race differences remained consistent in direction and statistically non-significant after adjusting for demographic and clinical characteristics (omnibus *p* = 0.263; Table 4 and Fig. 3). However, when considering individual adverse cardiovascular events, the rate of heart failure was statistically significant between races (*p* = 0.001), with the highest rate observed for hospitalizations in which the patient was Black (3.6%) and lowest when the patient was White (1.7%; see Table 3). Notably, between-race differences in adverse cardiovascular event rates were statistically similar across income quartiles (race-by-income quartile interaction *p* = 0.968; Fig. 4) suggesting that this adverse effect of race persists despite adjustment for income level.

**Table 3:** Unadjusted adverse event rates stratified by race

	White	Black	Hispanic	Other	*p*
Cardiovascular events	24.0 (22.7–25.3)	27.0 (24.1–30.1)	23.3 (21.0–25.9)	23.3 (20.3–26.6)	0.207
Heart failure	1.7 (1.4–2.1)	3.6 (2.6–5.1)	2.2 (1.5–3.2)	2.7 (1.8–4.2)	0.001
Arrhythmia	8.9 (8.2–9.7)	11.1 (9.2–13.3)	8.7 (7.3–10.2)	8.7 (6.8–11.0)	0.152
MI	*	*	*	*	-
Thromboembolic	17.2 (16.0–18.5)	17.6 (15.1–20.4)	16.4 (14.2–18.8)	15.6 (13.0–18.5)	0.651
Obstetric events	33.3 (32.1–34.6)	44.2 (14.1–47.3)	36.7 (34.1–39.4)	34.7 (31.4–38.2)	< .001
Hypertension in pregnancy	9.6 (8.9–10.4)	14.2 (12.1–16.5)	8.7 (7.3–10.4)	7.9 (6.1–10.2)	< .001
Placenta previa	2.3 (1.9–2.7)	3.2 (2.3–4.5)	1.5 (1.0–2.2)	3.4 (2.3–4.9)	0.006
Placental abruption	1.5 (1.2–1.8)	1.8 (1.2–2.9)	1.0 (0.6–1.7)	1.6 (0.9–2.8)	0.339
Hemorrhage	5.0 (4.5–5.6)	6.4 (5.0–8.2)	6.9 (5.7–8.3)	7.2 (5.6–9.3)	0.004
Pre-eclampsia	5.9 (5.3–6.5)	12.3 (10.4–14.6)	8.0 (6.7–9.5)	7.7 (6.0–10.0)	< .001
Pre-term delivery	7.9 (7.2–8.6)	14.1 (12.0–16.4)	9.7 (8.3–11.4)	9.5 (7.5–12.0)	< .001
Prolonged pregnancy	8.6 (7.9–9.4)	6.4 (5.0–8.1)	9.0 (7.7–10.6)	7.5 (5.8–9.6)	0.065
Fetal events	28.0 (26.9–29.3)	35.7 (32.7–38.9)	29.9 (27.6–32.2)	32.9 (29.6–36.4)	< .001
Distress	9.0 (8.3–9.7)	8.7 (7.1–10.6)	8.6 (7.3–10.2)	10.9 (8.9–13.4)	0.283
Growth restriction	4.7 (4.2–5.3)	8.0 (6.5–9.9)	4.4 (3.4–5.6)	6.0 (4.5–8.0)	< .001
Malformation	18.3 (17.3–19.4)	24.5 (21.9–27.4)	20.2 (18.2–22.5)	19.9 (17.1–23.0)	< .001
Death/stillbirth	0.9 (0.7–1.2)	1.2 (0.7–2.1)	1.1 (0.7–1.8)	1.5 (0.8–2.7)	0.332

Note: Data presented as percent (95% CI). An * indicates that the number of observed hospitalizations was 10 or less, which cannot be presented per the NIS Data Use Agreement

**Table 4:** Adjusted model results for adverse cardiovascular, obstetric, or fetal events

	Cardiovascular		Obstetric		Fetal	
	aOR (95% CI)	*p*	aOR (95% CI)	*p*	aOR (95% CI)	*p*
Race						
Black vs. White	1.05 (0.87–1.25)	0.632	1.36 (1.16–1.58)	< .001	1.31 (1.13–1.53)	< .001
Black vs. Hispanic	1.18 (0.95–1.46)	0.146	1.23 (1.03–1.47)	0.020	1.25 (1.04–1.49)	0.015
Black vs. Other	1.18 (0.93–1.50)	0.183	1.35 (1.09–1.67)	0.005	1.04 (0.84–1.28)	0.746
White vs. Hispanic	1.13 (0.96–1.32)	0.156	0.91 (0.80–1.04)	0.174	0.95 (0.83–1.09)	0.459
White vs. Other	1.13 (0.93–1.36)	0.217	1.00 (0.84–1.18)	0.971	0.79 (0.67–0.94)	0.007
Hispanic vs. Other	1.00 (0.80–1.26)	0.992	1.09 (0.89–1.34)	0.379	0.83 (0.69–1.01)	0.061
Age	Figure 3	< .001	Figure 3	0.629	Figure 3	0.109
Primary payer						
Medicaid	0.91 (0.73–1.12)	0.353	1.12 (0.91–1.37)	0.280	0.95 (0.79–1.15)	0.625
Private	0.83 (0.68–1.02)	0.078	1.05 (0.86–1.28)	0.660	0.97 (0.81–1.16)	0.708
Other	Reference		Reference		Reference	
Income quartile						
I	1.24 (1.05–1.46)	0.013	0.98 (0.85–1.13)	0.797	1.10 (0.95–1.27)	0.207
II	1.21 (1.04–1.41)	0.015	0.98 (0.85–1.12)	0.742	1.07 (0.94–1.23)	0.310
III	1.06 (0.91–1.23)	0.446	1.03 (0.90–1.17)	0.666	1.02 (0.90–1.17)	0.725
IV	Reference		Reference		Reference	
CHD category						
Severe	1.52 (1.21–1.90)	< .001	1.01 (0.83–1.22)	0.936	1.57 (1.29–1.91)	< .001
Shunt	1.04 (0.91–1.19)	0.606	0.92 (0.82–1.04)	0.178	0.90 (0.80–1.02)	0.104
Valve	1.22 (1.05–1.41)	0.008	0.95 (0.84–1.09)	0.470	0.91 (0.80–1.04)	0.183
Shunt + Valve	2.24 (1.59–3.16)	< .001	0.97 (0.69–1.36)	0.842	0.88 (0.62–1.23)	0.448
Other	Reference		Reference			
Congestive heart failure	9.01 (6.15–13.2)	< .001	1.20 (0.78–1.84)	0.416	0.90 (0.64–1.27)	0.555
Hypertension						
Systemic	1.16 (0.94–1.43)	0.173	34.1 (23.1–50.4)	< .001	1.20 (0.98–1.46)	0.076
Pulmonary	1.95 (1.50–2.53)	< .001	1.32 (1.01–1.74)	0.045	0.87 (0.67–1.13)	0.309
Diabetes	1.12 (0.80–1.58)	0.507	1.14 (0.80–1.63)	0.460	1.21 (0.89–1.66)	0.227

An estimated 35.1% (95% CI, 34.1 to 36.2%) of hospitalizations for delivery among those with CHD had an adverse obstetric event, with statistically higher rates observed for Black patients (44.2%) compared to Whites (33.3%), Hispanics (36.7%), and other races (34.7%; *p* < 0.001; Table 3 and Fig. 2); these differences were retained after adjusting for demographic and clinical characteristics (Table 4 and Fig. 3). When considering individual adverse obstetric events, between-race differences were driven by pregnancy-related hypertension, pre-eclampsia, and pre-term delivery (all *p* < 0.001; see Table 3). Race differences in obstetric adverse event rates were statistically similar across income quartiles (race-by-income quartile interaction *p* = 0.798; Fig. 4) suggesting that these racial differences persist despite adjusting for income differences.

An estimated 29.5% (95% CI, 28.6 to 30.5%) of CHD hospitalizations for delivery included an adverse fetal event, with statistically higher rates observed in Blacks (35.8%) compared to Whites (28.1%; *p* < 0.001) and Hispanics (29.9%; *p* = 0.003), but not for hospitalization in which the patient was of other race (32.9%; *p* = 0.224; Table 3 and Fig. 2); these differences were retained after adjusting for demographic and clinical characteristics (Table 4 and Fig. 3). Between-race differences were primarily driven by fetal growth restriction and malformation (both *p* < 0.001; see Table 3). Race differences in fetal adverse event rates differed across income quartiles (race-by-income quartile interaction *p* = 0.003; see supplemental Table S2; Fig. 4), with differences in fetal adverse event rates in Black compared to all other races being larger in the highest income quartile. This suggests that income differences do not completely explain the higher adverse fetal outcomes among Blacks.

The overall in-hospital mortality rate for hospitalizations with CHD was an estimated 0.1% (95% CI, 0.1 to 0.2%), which was statistically higher compared to those without a CHD diagnosis (0.008%, 95% CI, 0.007 to 0.008%, *p* < 0.001); race-specific mortality rates could not be reported due to the very low observed deaths per the NIS Data Use Agreement. Although statistically significant between-race differences were observed for length of stay (*p* < 0.001), the median stay for all races was 2 days.

## Discussion

The major findings of our study are as follows. (1) In pregnant women with CHD hospitalized for childbirth, Blacks had higher rates of obstetric and fetal adverse events. (2) While overall cardiovascular events were similar across races, Blacks had significantly higher rates of heart failure, and (3) the association between Black race and adverse events persisted even after adjustment for socioeconomic status. Health disparities are complex and the result of numerous social, environmental, biological, genetic, behavioral, healthcare delivery, and system or institutional factors. Our study demonstrated that Black patients with CHD had significantly higher rates of heart failure and adverse obstetric and fetal outcomes compared to Whites and Hispanics. This racial disparity was also noted in pregnancies without CHD, and our study findings are consistent with prior reports on race and pregnancy outcomes [6, 24–29]. However, our study highlights the fact that the adverse impact of race is much larger in magnitude in pregnant patients with CHD due to the high rate of adverse outcomes, i.e., the rate of adverse cardiovascular events was 3.0% vs. 2.6% respectively in Black vs. White patients without CHD (absolute difference of 0.4) compared to 27% vs. 24% respectively in Black vs. White pregnant women with CHD (absolute difference of 3.0%). Although the overall difference in cardiovascular events was not statistically different (likely due to the similar rates of thromboembolism which was the most common adverse event), rates of heart failure were notably higher for hospitalizations in which the patient was Black. This is not surprising given the higher rates of background hypertension in Black women. Furthermore, comorbidities like hypertension and diabetes are not just more prevalent but much less likely to be well-controlled in Black women [30, 31].

We also found that obstetric and fetal adverse events were higher in Black patients compared to White and Hispanic patients. In 2019, Schlichting et al. demonstrated the increased rates of maternal morbidity among women with CHD diagnoses [4]. Our study expands on their findings and additionally demonstrates that these adverse outcomes disproportionately affect pregnant Black women with CHD and highlight the disparities in outcomes among different racial/ethnic groups. The higher rates of maternal obstetric and fetal outcomes noted when the patient was Black could be due to a number of biologic (higher background comorbidities); socioeconomic (income, educational attainment); and/or care (healthcare access, systemic bias) factors. In our study, Blacks were younger, had a greater burden of hypertension, diabetes, more often belonged to lower-income quartiles, and carried Medicaid insurance which are concordant with prior observations [32, 33]. These are all known predictors of adverse maternal perinatal outcomes, such as postpartum hemorrhage, eclampsia, and adverse infant outcomes including preterm birth, poor fetal growth, low birth weight, and neonatal mortality [30–36]. Aside from this, care delivery itself might be different in hospitals that predominantly serve Black patients and this might be contributing to the adverse outcomes [25]. Similarly, the higher rates of adverse fetal outcomes in Black patients may be a direct consequence of the higher maternal obstetric events aside from all of the aforementioned biologic-, socioeconomic-, and care-related factors [29]. Preeclampsia and hypertension in pregnancy increase the risk of preterm delivery, which results in low-birth-weight infants. Higher rates of preterm delivery in Black women are likely related at least in part due to the elevated rates of pre-eclampsia [37, 38]. Our study findings are therefore consistent with prior reports on the impact of race on fetal and maternal outcomes though our study focused exclusively on women with CHD [15–17, 29].

A novel aspect that was explored in our study was the interaction between socioeconomic status (income quartile) and race in determining the outcomes of pregnant women with CHD. Although Black patients were more likely to belong to the lower income quartiles, there was no statistically significant interaction between race and income quartile with respect to maternal cardiovascular or obstetric outcomes. This implies that a higher income level does not negate the adverse outcomes of Black women with CHD and that these adverse outcomes may pertain to other factors which may include genetic or physiologic factors, access to healthcare/insurance, care delivery factors such as implicit or explicit bias as has been noted in other disease states such as hypertension [39–42]. Alternatively, this could be due to the fact that while the income quartile strongly correlates with socioeconomic status, it is not the sole determinant. Other factors like educational attainment, economic stability, employment, neighborhood, family, and community support may play a significant role in determining health care access and quality but are not necessarily captured by administrative databases like the NIS [43–45].

A number of limitations should be considered in the interpretation of our findings. First, this is a retrospective study using the NIS database which is an administrative database. Hence, coding inaccuracies can impact our sample identification and findings. Second, details of the exact CHD, lesion complexity, patient functional status, echocardiographic findings, and established predictors such as the World Health Organization (WHO) maternal risk score are unavailable and cannot be accounted for. Third, as previously mentioned, the income quartile may be a poor marker for socioeconomic status and may not capture many of the social determinants of health. Fourth, the outcomes assessed were limited to in-hospital outcomes given the nature of the NIS and we could not assess post-partum events that occur following discharge. Finally, because there are no linkages available between the records of the mother and the infant, fetal complications were only ascertained from those listed on the maternal record. As a result, the data on neonatal complications is likely incomplete. Despite these limitations, the NIS database is a well-validated dataset with stringent data accuracy checks and quality control. Our study is large and has the power to capture outcome differences that are not evident in single-center studies or smaller registries. Finally, data is ethnically and geographically diverse, includes a wide variety of centers and operators, and is likely much more representative of real-world practice and outcomes.

## Conclusion

In pregnant females with CHD admitted for delivery, the Black race is associated with a higher rate of obstetric complications and adverse fetal outcomes. While overall cardiovascular event rates did not differ by race, maternal rates of heart failure were higher among Blacks. Although a greater proportion of Black women were in lower income quartiles, the adverse impact of race on these outcomes was not attenuated even in Black women who belonged to the highest income quartile. Future research should focus on the specific mediators of this higher risk among Black women and implement specific strategies to mitigate the racial/ethnic disparities in pregnant women with CHD.

## Supplementary Information

Below is the link to the electronic supplementary material.

Supplementary file1 (DOCX 240 KB)

Supplementary file2 (DOCX 18 KB)

## Data Availability

Available for purchase from the HCUP family of databases sponsored by the Agency for Healthcare Research and Quality.

## Code Availability

Provided in supplementary Table 1.
