# Supplementary material for: Racial Disparities in Outcomes of Delivery and Cardiac Complications Among Pregnant Women with Congenital Heart Disease
Source: J Racial Ethn Health Disparities. 2024 Feb 28;12(2):1159–69. doi: 10.1007/s40615-024-01950-0 (PMC11913936; doi:10.1007/s40615-024-01950-0)
Supplement: Supplementary file 1 — Supplementary file1 (DOCX 240 KB) [file 40615_2024_1950_MOESM1_ESM.docx]

**Supplemental Table S1.** ICD-9/10 diagnosis and procedure codes

| **Congenital Heart Defects** | | | |
| --- | --- | --- | --- |
| **Category** | **Complication** | **ICD-9** | **ICD-10** |
| Congenital Heart Defects | Severe Defect | 745.0 | Q20.0 |
|  |  | 745.10 | Q20.3 |
|  |  | 745.10 | Q20.3 |
|  |  | 745.11 | Q20.1 |
|  |  | 745.12 | Q20.5 |
|  |  | 745.19 | Q20.3 |
|  |  |  | Q20.8 |
|  |  | 745.2 | Q21.3 |
|  |  | 745.3 | Q20.4 |
|  |  | 745.60 | Q21.2 |
|  |  | 745.60 | Q21.2 |
|  |  | 745.61 | Q21.2 |
|  |  | 745.69 | Q21.2 |
|  |  | 746.01 | Q22.0 |
|  |  | 746.1 | Q22.9 |
|  |  | 746.7 | Q23.4 |
|  |  | 747.11 | Q25.2 |
|  |  | 747.41 | Q26.2 |
|  | Shunt Defect | 745.4 | Q21.0 |
|  |  | 745.5 | Q21.1 |
|  |  | 745.8 | Q20.8 |
|  |  |  | Q21.8 |
|  |  | 745.9 | Q21.9 |
|  |  | 747.0 | Q25.0 |
|  |  | 747.1 | Q25.1 |
|  | Valve Defect | 746.00 | Q22.3 |
|  |  | 746.00 | Q22.3 |
|  |  | 746.02 | Q22.1 |
|  |  | 746.09 | Q22.2 |
|  |  | 746.2 | Q22.5 |
|  |  | 746.3 | Q23.0 |
|  |  | 746.4 | Q23.1 |
|  |  | 746.5 | Q23.2 |
|  |  | 746.6 | Q23.3 |
|  |  | 747.32 |  |
|  |  | 747.31 | Q25.5 |
|  |  |  | Q25.71 |
|  |  | 747.39 | Q25.6 |
|  |  |  | Q25.79 |
|  | Other Defect Only | 745.7 | Q20.8 |
|  |  | 746.81 | Q24.4 |
|  |  | 746.82 | Q24.2 |
|  |  | 746.83 | Q24.3 |
|  |  | 746.84 | Q24.8 |
|  |  | 746.85 | Q24.5 |
|  |  | 746.87 | Q24.0 |
|  |  |  | Q24.1 |
|  |  |  | Q24.8 |
|  |  | 746.89 | Q23.8 |
|  |  |  | Q24.8 |
|  |  | 746.9 | Q20.9 |
|  |  |  | Q24.9 |
|  |  | 747.29 | Q25.4x |
|  |  | 747.20 | Q25.4x |
|  |  | 747.21 | Q25.4x |
|  |  | 747.22 | Q25.2x |
|  |  |  | Q25.3 |
|  |  | 747.29 | Q25.4 |
|  |  | 747.40 | Q26.9 |
|  |  | 747.40 | Q26.9 |
|  |  | 747.42 | Q26.3 |
|  |  | 747.49 | Q26.0 |
|  |  |  | Q26.1 |
|  |  |  | Q26.8 |
|  |  | 747.9 | Q28.9 |
| **Adverse Events** | | | |
| Cardiovascular Events | Heart Failure | 4250 | I42.3 |
|  |  | 42500 |  |
|  |  | 4252 | I42.8 |
|  |  | 42520 |  |
|  |  | 4251 |  |
|  |  | 42510 |  |
|  |  | 4253 | I42.4 |
|  |  | 42530 |  |
|  |  | 4254 | I42.5 |
|  |  |  | I42.8 |
|  |  | 42540 |  |
|  |  | 4257 | I43 |
|  |  | 42570 |  |
|  |  | 4258 | I43 |
|  |  | 42580 |  |
|  |  | 4259 | I42.7 |
|  |  | 42590 |  |
|  |  | 42800 | I50.814 |
|  |  |  | I50.9 |
|  |  | 42810 | I50.1 |
|  |  | 42820 |  |
|  |  | 42821 | I50.21 |
|  |  | 42822 | I50.22 |
|  |  | 42823 | I50.23 |
|  |  | 42830 | I50.30 |
|  |  | 42831 | I50.31 |
|  |  | 42832 | I50.32 |
|  |  | 42833 | I50.33 |
|  |  | 42840 | I50.40 |
|  |  | 42841 | I50.41 |
|  |  | 42842 | I50.42 |
|  |  | 42843 | I50.43 |
|  |  | 42890 | I50.810 |
|  |  |  | I50.811 |
|  |  |  | I50.812 |
|  |  |  | I50.813 |
|  |  |  | I50.82 |
|  |  |  | I50.83 |
|  |  |  | I50.84 |
|  |  |  | I50.89 |
|  |  |  | I50.9 |
|  |  | 39891 | I09.81 |
|  |  | 67450 | O90.3 |
|  |  | 67451 | O90.3 |
|  |  | 67452 | O90.3 |
|  |  | 67453 | O90.3 |
|  |  | 67454 | O90.3 |
|  | Arrhythmia | 4270 | I47.1 |
|  |  | 42700 |  |
|  |  | 4271 | I47.2 |
|  |  | 42710 |  |
|  |  | 4272 | I47.9 |
|  |  | 42720 |  |
|  |  | 4279 | I49.9 |
|  |  | 42790 |  |
|  |  | 7850 | R00.0 |
|  |  | 78500 |  |
|  |  | 7851 | R00.2 |
|  |  | 78510 |  |
|  |  | 42731 | I48.91 |
|  |  | 42732 | I48.92 |
|  |  | 42760 | I49.40 |
|  |  | 42761 | I49.1 |
|  |  | 42769 | I49.3 |
|  |  |  | I49.49 |
|  |  | 42781 | I49.5 |
|  |  |  | R00.1 |
|  |  | 42789 | I49.8 |
|  |  |  | R00.1 |
|  |  | 4275 | I46.9 |
|  |  | 42750 |  |
|  |  | 42741 | I49.01 |
|  |  | 42742 | I49.02 |
|  |  | V1253 | Z86.74 |
|  |  | 4260 | I44.2 |
|  |  | 42600 |  |
|  |  | 4262 | I44.4 |
|  |  |  | I44.5 |
|  |  |  | I44.60 |
|  |  |  | I44.69 |
|  |  | 42620 |  |
|  |  | 4263 | I44.7 |
|  |  | 42630 |  |
|  |  | 4264 | I45.10 |
|  |  | 42640 |  |
|  |  | 4266 | I45.5 |
|  |  | 42660 |  |
|  |  | 4267 | I45.6 |
|  |  | 42670 |  |
|  |  | 4269 | I45.9 |
|  |  | 42690 |  |
|  |  | 42610 | I44.30 |
|  |  | 42611 | I44.0 |
|  |  | 42612 | I44.1 |
|  |  | 42613 | I44.2 |
|  |  | 42650 | I44.30 |
|  |  |  | I44.39 |
|  |  |  | I45.4 |
|  |  | 42651 | I45.2 |
|  |  | 42652 | I45.2 |
|  |  | 42653 | I45.2 |
|  |  | 42654 | I45.3 |
|  |  | 42681 | I45.6 |
|  |  | 42682 | I45.81 |
|  |  | 42689 | I45.89 |
|  |  | V450 |  |
|  |  | V4500 | Z95.9 |
|  |  | V4501 | Z95.0 |
|  |  | V4502 | Z95.810 |
|  |  | V4509 | Z95.818 |
|  |  | V533 |  |
|  |  | V5331 | Z45.010 |
|  |  |  | Z45.018 |
|  |  | V5332 | Z45.02 |
|  |  | V5339 | Z45.09 |
|  | Myocardial Infarction | 41000 | I21.09 |
|  |  | 41001 | I21.09 |
|  |  | 41002 | I21.09 |
|  |  | 41010 | I21.09 |
|  |  | 41011 | I21.09 |
|  |  | 41012 | I21.09 |
|  |  | 41020 | I21.19 |
|  |  | 41021 | I21.19 |
|  |  | 41022 | I21.19 |
|  |  | 41030 | I21.11 |
|  |  | 41031 | I21.11 |
|  |  | 41032 | I21.11 |
|  |  | 41040 | I21.19 |
|  |  | 41041 | I21.19 |
|  |  | 41042 | I21.19 |
|  |  | 41050 | I21.29 |
|  |  | 41051 | I21.29 |
|  |  | 41052 | I21.29 |
|  |  | 41060 | I21.29 |
|  |  | 41061 | I21.29 |
|  |  | 41062 | I21.29 |
|  |  | 41070 | I21.4 |
|  |  | 41071 | I21.4 |
|  |  | 41072 | I21.4 |
|  |  | 41080 | I21.29 |
|  |  | 41081 | I21.29 |
|  |  | 41082 | I21.29 |
|  |  | 41090 | I21.3 |
|  |  |  | I21.9 |
|  |  |  | I21.A1 |
|  |  |  | I21.A9 |
|  |  | 41091 | I21.3 |
|  |  |  | I21.9 |
|  |  |  | I21.A1 |
|  |  |  | I21.A9 |
|  |  | 41092 | I21.3 |
|  |  |  | I21.9 |
|  |  |  | I21.A1 |
|  |  |  | I21.A9 |
|  |  | 41100 | I24.1 |
|  |  | 41110 | I20.0 |
|  |  | 41180 |  |
|  | Thromboembolic event | 64930 | O99.119 |
|  |  | 64931 | O99.111 |
|  |  |  | O99.112 |
|  |  |  | O99.113 |
|  |  |  | O99.12 |
|  |  | 64932 | O99.13 |
|  |  | 64933 | O99.111 |
|  |  |  | O99.112 |
|  |  |  | O99.113 |
|  |  | 64934 | O99.13 |
|  |  | 67120 | O22.20 |
|  |  | 67121 | O22.21 |
|  |  |  | O22.22 |
|  |  |  | O22.23 |
|  |  | 67122 | O87.0 |
|  |  | 67123 | O22.21 |
|  |  |  | O22.22 |
|  |  |  | O22.23 |
|  |  | 67124 | O87.0 |
|  |  | 67130 | O22.30 |
|  |  | 67131 | O22.31 |
|  |  |  | O22.32 |
|  |  |  | O22.33 |
|  |  | 67133 | O22.31 |
|  |  |  | O22.32 |
|  |  |  | O22.33 |
|  |  | 67140 | O87.1 |
|  |  | 67142 | O87.1 |
|  |  | 67144 | O87.1 |
|  |  | 67150 | O22.50 |
|  |  | 67151 | O22.51 |
|  |  |  | O22.52 |
|  |  |  | O22.53 |
|  |  |  | O22.91 |
|  |  |  | O22.92 |
|  |  |  | O22.93 |
|  |  | 67152 | O87.3 |
|  |  | 67153 | O22.51 |
|  |  |  | O22.52 |
|  |  |  | O22.53 |
|  |  |  | O22.91 |
|  |  |  | O22.92 |
|  |  |  | O22.93 |
|  |  | 67154 | O87.3 |
|  |  | 67180 | O22.40 |
|  |  |  | O22.8X9 |
|  |  | 67181 | O22.41 |
|  |  |  | O22.42 |
|  |  |  | O22.43 |
|  |  |  | O22.8X1 |
|  |  |  | O22.8X2 |
|  |  |  | O22.8X3 |
|  |  | 67182 | O87.2 |
|  |  |  | O87.8 |
|  |  | 67183 | O22.41 |
|  |  |  | O22.42 |
|  |  |  | O22.43 |
|  |  |  | O22.8X1 |
|  |  |  | O22.8X2 |
|  |  |  | O22.8X3 |
|  |  | 67184 | O87.2 |
|  |  |  | O87.8 |
|  |  | 67190 | O22.90 |
|  |  | 67191 | O22.91 |
|  |  |  | O22.92 |
|  |  |  | O22.93 |
|  |  | 67192 | O87.9 |
|  |  | 67193 | O22.91 |
|  |  |  | O22.92 |
|  |  |  | O22.93 |
|  |  | 67194 | O87.9 |
|  |  | 67320 | O88.219 |
|  |  | 67321 | O88.211 |
|  |  |  | O88.212 |
|  |  |  | O88.213 |
|  |  |  | O88.22 |
|  |  | 67322 | O88.22 |
|  |  |  | O88.23 |
|  |  | 67323 | O88.211 |
|  |  |  | O88.212 |
|  |  |  | O88.213 |
|  |  | 67324 | O88.23 |
|  |  | 67381 | O88.811 |
|  |  |  | O88.812 |
|  |  |  | O88.813 |
|  |  |  | O88.82 |
|  |  | 67382 | O88.811 |
|  |  |  | O88.812 |
|  |  |  | O88.813 |
|  |  |  | O88.82 |
|  |  |  | O88.83 |
|  |  | 67383 | O88.811 |
|  |  |  | O88.812 |
|  |  |  | O88.813 |
|  |  | 67384 | O88.83 |
|  |  | 67400 | O99.419 |
|  |  | 67401 | O99.411 |
|  |  |  | O99.412 |
|  |  |  | O99.413 |
|  |  |  | O99.42 |
|  |  | 67402 | O99.42 |
|  |  |  | O99.43 |
|  |  | 67403 | O99.411 |
|  |  |  | O99.412 |
|  |  |  | O99.413 |
|  |  | 67404 | O99.43 |
| Obstetric Events | Hypertension in pregnancy | 64230 | O13.9 |
|  |  | 64231 | O13.1 |
|  |  |  | O13.2 |
|  |  |  | O13.3 |
|  |  |  | O13.4 |
|  |  |  | O16.1 |
|  |  |  | O16.2 |
|  |  |  | O16.3 |
|  |  | 64232 | O13.1 |
|  |  |  | O13.2 |
|  |  |  | O13.3 |
|  |  |  | O13.5 |
|  |  | 64233 | O13.1 |
|  |  |  | O13.2 |
|  |  |  | O13.3 |
|  |  |  | O16.1 |
|  |  |  | O16.2 |
|  |  |  | O16.3 |
|  |  | 64234 | O13.1 |
|  |  |  | O13.2 |
|  |  |  | O13.3 |
|  |  |  | O13.5 |
|  |  | 64290 | O16.9 |
|  |  | 64291 | O16.1 |
|  |  |  | O16.2 |
|  |  |  | O16.3 |
|  |  |  | O16.4 |
|  |  | 64292 | O16.5 |
|  |  |  | O16.9 |
|  |  | 64293 | O16.1 |
|  |  |  | O16.2 |
|  |  |  | O16.3 |
|  |  | 64294 | O16.1 |
|  |  |  | O16.2 |
|  |  |  | O16.3 |
|  |  |  | O16.5 |
|  |  | 64200 | O10.019 |
|  |  |  | O10.919 |
|  |  | 64201 | O10.011 |
|  |  |  | O10.012 |
|  |  |  | O10.013 |
|  |  |  | O10.02 |
|  |  |  | O10.911 |
|  |  |  | O10.912 |
|  |  |  | O10.913 |
|  |  |  | O10.92 |
|  |  | 64202 | O10.03 |
|  |  | 64203 | O10.011 |
|  |  |  | O10.012 |
|  |  |  | O10.013 |
|  |  |  | O10.911 |
|  |  |  | O10.912 |
|  |  |  | O10.913 |
|  |  | 64204 | O10.03 |
|  |  |  | O10.93 |
|  |  | 64210 | O10.419 |
|  |  | 64211 | O10.411 |
|  |  |  | O10.412 |
|  |  |  | O10.413 |
|  |  |  | O10.42 |
|  |  | 64212 | O10.43 |
|  |  | 64213 | O10.411 |
|  |  |  | O10.412 |
|  |  |  | O10.413 |
|  |  | 64214 | O10.43 |
|  |  | 64220 | O10.119 |
|  |  |  | O10.219 |
|  |  |  | O10.319 |
|  |  |  | O11.9 |
|  |  | 64221 | O10.111 |
|  |  |  | O10.112 |
|  |  |  | O10.113 |
|  |  |  | O10.12 |
|  |  |  | O10.211 |
|  |  |  | O10.212 |
|  |  |  | O10.213 |
|  |  |  | O10.22 |
|  |  |  | O10.311 |
|  |  |  | O10.312 |
|  |  |  | O10.313 |
|  |  |  | O10.32 |
|  |  |  | O11.1 |
|  |  |  | O11.2 |
|  |  |  | O11.3 |
|  |  | 64222 | O10.13 |
|  |  | 64223 | O10.111 |
|  |  |  | O10.112 |
|  |  |  | O10.113 |
|  |  |  | O10.211 |
|  |  |  | O10.212 |
|  |  |  | O10.213 |
|  |  |  | O10.311 |
|  |  |  | O10.312 |
|  |  |  | O10.313 |
|  |  |  | O11.1 |
|  |  |  | O11.2 |
|  |  |  | O11.3 |
|  |  | 64224 | O10.13 |
|  |  |  | O10.23 |
|  |  |  | O10.33 |
|  |  | 7600 | P00.0 |
|  |  | 76000 |  |
|  | Placenta Previa | 64100 | O44.00 |
|  |  |  | O44.20 |
|  |  |  | O44.40 |
|  |  | 64101 | O44.01 |
|  |  |  | O44.02 |
|  |  |  | O44.03 |
|  |  |  | O44.21 |
|  |  |  | O44.22 |
|  |  |  | O44.23 |
|  |  |  | O44.41 |
|  |  |  | O44.42 |
|  |  |  | O44.43 |
|  |  | 64103 | O44.01 |
|  |  |  | O44.02 |
|  |  |  | O44.03 |
|  |  |  | O44.21 |
|  |  |  | O44.22 |
|  |  |  | O44.23 |
|  |  |  | O44.41 |
|  |  |  | O44.42 |
|  |  |  | O44.43 |
|  |  | 64110 | O44.10 |
|  |  |  | O44.30 |
|  |  |  | O44.50 |
|  |  | 64111 | O44.11 |
|  |  |  | O44.12 |
|  |  |  | O44.13 |
|  |  |  | O44.31 |
|  |  |  | O44.32 |
|  |  |  | O44.33 |
|  |  |  | O44.51 |
|  |  |  | O44.52 |
|  |  |  | O44.53 |
|  |  | 64113 | O44.11 |
|  |  |  | O44.12 |
|  |  |  | O44.13 |
|  |  |  | O44.31 |
|  |  |  | O44.32 |
|  |  |  | O44.33 |
|  |  |  | O44.51 |
|  |  |  | O44.52 |
|  |  |  | O44.53 |
|  |  | 64120 | O45.8X9 |
|  |  | 64121 | O45.8X1 |
|  |  |  | O45.8X2 |
|  |  |  | O45.8X3 |
|  |  |  | O45.91 |
|  |  |  | O45.92 |
|  |  |  | O45.93 |
|  |  | 64123 | O45.8X1 |
|  |  |  | O45.8X2 |
|  |  |  | O45.8X3 |
|  |  |  | O45.91 |
|  |  |  | O45.92 |
|  |  |  | O45.93 |
|  | Placental abruption | 64120 | O45.8X9 |
|  |  | 64121 | O45.8X1 |
|  |  |  | O45.8X2 |
|  |  |  | O45.8X3 |
|  |  |  | O45.91 |
|  |  |  | O45.92 |
|  |  |  | O45.93 |
|  |  | 64123 | O45.8X1 |
|  |  |  | O45.8X2 |
|  |  |  | O45.8X3 |
|  |  |  | O45.91 |
|  |  |  | O45.92 |
|  |  |  | O45.93 |
|  | Hemorrhage | 66600 | O72.0 |
|  |  | 66602 | O72.0 |
|  |  |  | O43.211 |
|  |  |  | O43.212 |
|  |  |  | O43.213 |
|  |  |  | O43.221 |
|  |  |  | O43.222 |
|  |  |  | O43.223 |
|  |  |  | O43.231 |
|  |  |  | O43.232 |
|  |  |  | O43.233 |
|  |  | 66604 | O72.0 |
|  |  |  | O43.211 |
|  |  |  | O43.212 |
|  |  |  | O43.213 |
|  |  |  | O43.221 |
|  |  |  | O43.222 |
|  |  |  | O43.223 |
|  |  |  | O43.231 |
|  |  |  | O43.232 |
|  |  |  | O43.233 |
|  |  | 66610 | O72.1 |
|  |  | 66612 | O72.1 |
|  |  | 66614 | O72.1 |
|  |  | 66620 | O72.2 |
|  |  | 66622 | O72.2 |
|  |  | 66624 | O72.2 |
|  |  | 66630 | O72.3 |
|  |  | 66632 | O72.3 |
|  |  | 66634 | O72.3 |
|  |  | 64000 | O20.0 |
|  |  | 64001 | O20.0 |
|  |  | 64003 | O20.0 |
|  |  | 64080 | O20.8 |
|  |  | 64081 | O20.8 |
|  |  | 64083 | O20.8 |
|  |  | 64090 | O20.9 |
|  |  | 64091 | O20.9 |
|  |  | 64093 | O20.9 |
|  |  | 64130 | O46.009 |
|  |  |  | O46.019 |
|  |  |  | O46.029 |
|  |  |  | O46.099 |
|  |  | 64131 | O45.001 |
|  |  |  | O45.002 |
|  |  |  | O45.003 |
|  |  |  | O45.011 |
|  |  |  | O45.012 |
|  |  |  | O45.013 |
|  |  |  | O45.021 |
|  |  |  | O45.022 |
|  |  |  | O45.023 |
|  |  |  | O45.091 |
|  |  |  | O45.092 |
|  |  |  | O45.093 |
|  |  |  | O46.001 |
|  |  |  | O46.002 |
|  |  |  | O46.003 |
|  |  |  | O46.011 |
|  |  |  | O46.012 |
|  |  |  | O46.013 |
|  |  |  | O46.021 |
|  |  |  | O46.022 |
|  |  |  | O46.023 |
|  |  |  | O45.091 |
|  |  |  | O45.092 |
|  |  |  | O45.093 |
|  |  |  | O67.0 |
|  |  | 64133 | O45.001 |
|  |  |  | O45.002 |
|  |  |  | O45.003 |
|  |  |  | O45.011 |
|  |  |  | O45.012 |
|  |  |  | O45.013 |
|  |  |  | O45.021 |
|  |  |  | O45.022 |
|  |  |  | O45.023 |
|  |  |  | O45.091 |
|  |  |  | O45.092 |
|  |  |  | O45.093 |
|  |  |  | O46.001 |
|  |  |  | O46.002 |
|  |  |  | O46.003 |
|  |  |  | O46.011 |
|  |  |  | O46.012 |
|  |  |  | O46.013 |
|  |  |  | O46.021 |
|  |  |  | O46.022 |
|  |  |  | O46.023 |
|  |  |  | O45.091 |
|  |  |  | O45.092 |
|  |  |  | O45.093 |
|  |  | 64180 | O46.8X9 |
|  |  | 64181 | O46.8X1 |
|  |  |  | O46.8X2 |
|  |  |  | O46.8X3 |
|  |  |  | O67.8 |
|  |  | 64183 | O46.8X1 |
|  |  |  | O46.8X2 |
|  |  |  | O46.8X3 |
|  |  | 64190 | O46.90 |
|  |  | 64191 | O46.91 |
|  |  |  | O46.92 |
|  |  |  | O46.93 |
|  |  |  | O67.9 |
|  |  | 64193 | O46.91 |
|  |  |  | O46.92 |
|  |  |  | O46.93 |
|  | Pre-eclampsia | 64240 | O14.00 |
|  |  |  | O14.90 |
|  |  | 64241 | O14.02 |
|  |  |  | O14.03 |
|  |  |  | O14.04 |
|  |  |  | O14.92 |
|  |  |  | O14.93 |
|  |  |  | O14.94 |
|  |  | 64242 | O14.02 |
|  |  |  | O14.03 |
|  |  |  | O14.05 |
|  |  |  | O14.95 |
|  |  | 64243 | O14.02 |
|  |  |  | O14.03 |
|  |  |  | O14.92 |
|  |  |  | O14.93 |
|  |  | 64244 | O14.05 |
|  |  |  | O14.95 |
|  |  |  | O15.2 |
|  |  | 64250 | O14.10 |
|  |  |  | O14.20 |
|  |  | 64251 | O14.12 |
|  |  |  | O14.13 |
|  |  |  | O14.14 |
|  |  |  | O14.22 |
|  |  |  | O14.23 |
|  |  |  | O14.24 |
|  |  | 64252 | O14.12 |
|  |  |  | O14.13 |
|  |  |  | O14.14 |
|  |  |  | O14.22 |
|  |  |  | O14.23 |
|  |  |  | O14.24 |
|  |  | 64253 | O14.12 |
|  |  |  | O14.13 |
|  |  |  | O14.22 |
|  |  |  | O14.23 |
|  |  | 64254 | O14.12 |
|  |  |  | O14.13 |
|  |  |  | O14.15 |
|  |  |  | O14.22 |
|  |  |  | O14.23 |
|  |  |  | O14.25 |
|  |  | 64260 | O15.9 |
|  |  | 64261 | O15.02 |
|  |  |  | O15.03 |
|  |  |  | O15.1 |
|  |  | 64262 | O15.2 |
|  |  | 64263 | O15.02 |
|  |  |  | O15.03 |
|  |  | 64264 | O15.2 |
|  |  | 64270 | O11.9 |
|  |  | 64271 | O11.1 |
|  |  |  | O11.2 |
|  |  |  | O11.3 |
|  |  |  | O11.4 |
|  |  | 64272 | O11.4 |
|  |  | 64273 | O11.1 |
|  |  |  | O11.2 |
|  |  |  | O11.3 |
|  |  | 64274 | O11.5 |
|  | Preterm delivery | 64420 | O60.10X0 |
|  |  | 64421 | O60.12X0 |
|  |  |  | O60.13X0 |
|  |  |  | O60.14X0 |
|  |  | 76400 | P05.00 |
|  |  |  | P05.09 |
|  |  |  | P05.10 |
|  |  |  | P05.19 |
|  |  | 76401 | P05.01 |
|  |  |  | P05.11 |
|  |  | 76402 | P05.02 |
|  |  |  | P05.12 |
|  |  | 76403 | P05.03 |
|  |  |  | P05.13 |
|  |  | 76404 | P05.04 |
|  |  |  | P05.14 |
|  |  | 76405 | P05.05 |
|  |  |  | P05.15 |
|  |  | 76406 | P05.06 |
|  |  |  | P05.16 |
|  |  | 76407 | P05.07 |
|  |  |  | P05.17 |
|  |  | 76408 | P05.08 |
|  |  |  | P05.18 |
|  |  | 7650 |  |
|  |  | 76500 | P07.00 |
|  |  |  | P07.10 |
|  |  | 76501 | P07.01 |
|  |  | 76502 | P07.02 |
|  |  | 76503 | P07.03 |
|  |  | 76504 | P07.14 |
|  |  | 76505 | P07.15 |
|  |  | 76506 | P07.16 |
|  |  | 76507 | P07.17 |
|  |  | 76508 | P07.18 |
|  |  | 76509 | P07.30 |
|  |  | 7651 |  |
|  |  | 76510 | P07.00 |
|  |  |  | P07.10 |
|  |  | 76511 | P07.01 |
|  |  | 76512 | P07.02 |
|  |  | 76513 | P07.03 |
|  |  | 76514 | P07.14 |
|  |  | 76515 | P07.15 |
|  |  | 76516 | P07.16 |
|  |  | 76517 | P07.17 |
|  |  | 76518 | P07.18 |
|  |  | 76519 | P07.30 |
|  |  | 76521 | P07.21 |
|  |  |  | P07.22 |
|  |  | 76522 | P07.23 |
|  |  | 76523 | P07.24 |
|  |  |  | P07.25 |
|  |  | 76524 | P07.26 |
|  |  |  | P07.31 |
|  |  | 76525 | P07.32 |
|  |  |  | P07.33 |
|  |  | 76526 | P07.34 |
|  |  |  | P07.35 |
|  |  | 76527 | P07.36 |
|  |  |  | P07.37 |
|  |  | 76528 | P07.38 |
|  |  |  | P07.39 |
|  |  | V2131 | P07.01 |
|  |  | V2132 | P07.02 |
|  |  |  | P07.03 |
|  |  | V2133 | P07.14 |
|  |  |  | P07.15 |
|  |  | V2134 | P07.16 |
|  |  |  | P07.17 |
|  |  | V2135 | P07.18 |
|  |  | 7742 | P59.0 |
|  | Prolonged Pregnancy | 64500 |  |
|  |  | 64501 |  |
|  |  | 64503 |  |
|  |  | 64510 | O48.0 |
|  |  | 64511 | O48.0 |
|  |  | 64513 | O48.0 |
|  |  | 64520 | O48.1 |
|  |  | 64521 | O48.1 |
|  |  | 64523 | O48.1 |
| Fetal Events | Fetal distress | 65630 | O68 |
|  |  | 65631 | O68 |
|  |  | 65633 | O68 |
|  |  | 66100 | O62.0 |
|  |  | 66101 | O62.0 |
|  |  | 66103 | O62.0 |
|  |  | 66110 | O62.1 |
|  |  | 66111 | O62.1 |
|  |  | 66113 | O62.1 |
|  |  | 66120 | O62.2 |
|  |  | 66121 | O62.2 |
|  |  | 66123 | O62.2 |
|  |  | 66130 | O62.3 |
|  |  | 66131 | O62.3 |
|  |  | 66133 | O62.3 |
|  |  | 66140 | O62.4 |
|  |  | 66141 | O62.4 |
|  |  | 66143 | O62.4 |
|  |  | 66190 | O62.9 |
|  |  | 66191 | O62.9 |
|  |  | 66193 | O62.9 |
|  |  | 66200 | O63.0 |
|  |  | 66201 | O63.0 |
|  |  | 66203 | O63.0 |
|  |  | 66210 | O63.9 |
|  |  | 66211 | O63.9 |
|  |  | 66213 | O63.9 |
|  |  | 66220 | O63.1 |
|  |  | 66221 | O63.1 |
|  |  | 66223 | O63.1 |
|  |  | 66230 | O63.2 |
|  |  | 66231 | O63.2 |
|  |  | 66233 | O63.2 |
|  | Fetal Growth Restriction | 65650 | O36.5190 |
|  |  |  | O36.5990 |
|  |  | 65651 | O36.5110 |
|  |  |  | O36.5120 |
|  |  |  | O36.5130 |
|  |  |  | O36.5910 |
|  |  |  | O36.5920 |
|  |  |  | O36.5930 |
|  |  | 65653 | O36.5110 |
|  |  |  | O36.5120 |
|  |  |  | O36.5130 |
|  |  |  | O36.5910 |
|  |  |  | O36.5920 |
|  |  |  | O36.5930 |
|  |  | 76409 | P05.09 |
|  |  | 76410 | P05.00 |
|  |  |  | P05.10 |
|  |  | 76411 | P05.01 |
|  |  |  | P05.11 |
|  |  | 76412 | P05.02 |
|  |  |  | P05.12 |
|  |  | 76413 | P05.03 |
|  |  |  | P05.13 |
|  |  | 76414 | P05.04 |
|  |  |  | P05.14 |
|  |  | 76415 | P05.05 |
|  |  |  | P05.15 |
|  |  | 76416 | P05.06 |
|  |  |  | P05.16 |
|  |  | 76417 | P05.07 |
|  |  |  | P05.17 |
|  |  | 76418 | P05.08 |
|  |  |  | P05.18 |
|  |  | 76419 | P05.09 |
|  |  | 76420 | P05.2 |
|  |  | 76421 | P05.2 |
|  |  | 76422 | P05.2 |
|  |  | 76423 | P05.2 |
|  |  | 76424 | P05.2 |
|  |  | 76425 | P05.2 |
|  |  | 76426 | P05.2 |
|  |  | 76427 | P05.2 |
|  |  | 76428 | P05.2 |
|  |  | 76429 | P05.2 |
|  |  | 76490 | P05.9 |
|  |  | 76491 | P05.9 |
|  |  | 76492 | P05.9 |
|  |  | 76493 | P05.9 |
|  |  | 76494 | P05.9 |
|  |  | 76495 | P05.9 |
|  |  | 76496 | P05.9 |
|  |  | 76497 | P05.9 |
|  |  | 76498 | P05.9 |
|  |  | 76499 | P05.9 |
|  |  | V2130 | P07.10 |
|  | Fetal Malformation | 65500 | O35.0XX0 |
|  |  | 65501 | O35.0XX0 |
|  |  | 65503 | O35.0XX0 |
|  |  | 65510 | O35.1XX0 |
|  |  | 65511 | O35.1XX0 |
|  |  | 65513 | O35.1XX0 |
|  |  | 65580 | O35.8XX0 |
|  |  | 65581 | O35.8XX0 |
|  |  | 65583 | O35.8XX0 |
|  |  | 65590 | O35.9XX0 |
|  |  | 65591 | O35.9XX0 |
|  |  | 65593 | O35.9XX0 |
|  |  | 65970 | O76 |
|  |  | 65971 | O76 |
|  |  | 65973 | O76 |
|  | Fetal death or stillbirth | V271 | Z37.1 |
|  |  | V273 | Z37.3 |
|  |  | V274 | Z37.4 |
|  |  | V276 | Z37.69 |
|  |  | V277 | Z37.7 |
|  |  | 65640 | O36.4XX0 |
|  |  | 65641 | O36.4XX0 |
|  |  | 65643 | O36.4XX0 |
|  |  | 7680 | P84 |
|  |  | 7681 | P84 |
| Delivery Method (procedure codes) | Artifical rupture of the membranes | 730 |  |
|  |  | 7301 | 10900ZC |
|  |  |  | 10903ZC |
|  |  |  | 10904ZC |
|  |  |  | 10907ZC |
|  |  |  | 10908ZC |
|  |  | 7309 | 10907ZC |
|  |  |  | 10908ZC |
|  | Cesarean Section | DRG:765-766 |  |
|  |  | 74.0-74.99 except for 74.91 | 10D00Z0 |
|  |  |  | 10D00Z1 |
|  |  |  | 10D00Z2 |
|  |  |  | 10T20ZZ |
|  |  |  | 10T23ZZ |
|  |  |  | 10T24ZZ |
|  |  |  | 10D00Z0 |
|  | Induction | 7301 | 10900ZC |
|  |  |  | 10903ZC |
|  |  |  | 10904ZC |
|  |  |  | 10907ZC |
|  |  |  | 10908ZC |
|  |  | 731 | 0U7C7ZZ |
|  |  | 734 | 3E030VJ |
|  |  |  | 3E033VJ |
|  |  |  | 3E040VJ |
|  |  |  | 3E043VJ |
|  |  |  | 3E050VJ |
|  |  |  | 3E053VJ |
|  |  |  | 3E060VJ |
|  |  |  | 3E063VJ |
|  |  |  | 3E0DXGC |
|  |  |  | 3E0P3VZ |
|  |  |  | 3E0P7VZ |
|  |  | 9649 | 0UH37YZ |
|  |  |  | 0UH38YZ |
|  |  |  | 0UH87YZ |
|  |  |  | 0UH88YZ |
|  |  |  | 0UHH7YZ |
|  |  |  | 0UHH8YZ |
|  |  |  | 0VH47YZ |
|  |  |  | 0VH48YZ |
|  |  |  | 0VH87YZ |
|  |  |  | 0VH88YZ |
|  |  |  | 0VHD7YZ |
|  |  |  | 0VHD8YZ |
|  |  |  | 0VHM7YZ |
|  |  |  | 0VHM8YZ |
|  |  |  | 0VHR7YZ |
|  |  |  | 0VHR8YZ |
|  |  |  | 0WHR73Z |
|  |  |  | 0WHR7YZ |
|  |  |  | 10A07ZX |
|  |  |  | 3E1K78Z |
|  |  |  | 3E1K88Z |
|  | operative vaginal delivery | 720 | 10D07Z3 |
|  |  | 721 | 0W8NXZZ |
|  |  |  | 10D07Z3 |
|  |  | 722 |  |
|  |  | 7221 | 0W8NXZZ |
|  |  |  | 10D07Z4 |
|  |  | 7229 | 10D07Z4 |
|  |  | 723 |  |
|  |  | 7231 | 0W8NXZZ |
|  |  |  | 10D07Z5 |
|  |  | 7239 | 10D07Z5 |
|  |  | 724 | 10S07ZZ |
|  |  | 725 | 10D07Z3 |
|  |  |  | 10D07Z4 |
|  |  |  | 10D07Z5 |
|  |  | 7251 | 10D07Z3 |
|  |  |  | 10D07Z4 |
|  |  |  | 10D07Z5 |
|  |  | 7252 | 10D07Z6 |
|  |  | 7253 | 10D07Z3 |
|  |  |  | 10D07Z4 |
|  |  |  | 10D07Z5 |
|  |  | 7254 | 10D07Z6 |
|  |  | 726 | 10D07Z3 |
| **Comorbidities** | | | |
| **Category** | **Comorbidity** | **ICD-9** | **ICD-10** |
| Comorbidity | Hypertension | CM_HTN_C | |
|  | Heart Failure | CM_CHF | |
|  | Pulmonary Hypertension | CM_PULMCIRC | |
|  | Coronary Artery Disease | 4100 |  |
|  |  | 41000 | I21.09 |
|  |  | 41001 | I21.09 |
|  |  | 41002 | I21.09 |
|  |  | 4101 |  |
|  |  | 41010 | I21.09 |
|  |  | 41011 | I21.09 |
|  |  | 41012 | I21.09 |
|  |  | 4102 |  |
|  |  | 41020 | I21.19 |
|  |  | 41021 | I21.19 |
|  |  | 41022 | I21.19 |
|  |  | 4103 |  |
|  |  | 41030 | I21.11 |
|  |  | 41031 | I21.11 |
|  |  | 41032 | I21.11 |
|  |  | 4104 |  |
|  |  | 41040 | I21.19 |
|  |  | 41041 | I21.19 |
|  |  | 41042 | I21.19 |
|  |  | 4105 |  |
|  |  | 41050 | I21.29 |
|  |  | 41051 | I21.29 |
|  |  | 41052 | I21.29 |
|  |  | 4106 |  |
|  |  | 41060 | I21.29 |
|  |  | 41061 | I21.29 |
|  |  | 41062 | I21.29 |
|  |  | 4107 |  |
|  |  | 41070 | I21.4 |
|  |  | 41071 | I21.4 |
|  |  | 41072 | I21.4 |
|  |  | 4108 |  |
|  |  | 41080 | I21.29 |
|  |  | 41081 | I21.29 |
|  |  | 41082 | I21.29 |
|  |  | 4109 |  |
|  |  | 41090 | I21.3 |
|  |  |  | I21.9 |
|  |  |  | I21.A1 |
|  |  |  | I21.A9 |
|  |  | 41091 | I21.3 |
|  |  |  | I21.9 |
|  |  |  | I21.A1 |
|  |  |  | I21.A9 |
|  |  | 41092 | I21.3 |
|  |  |  | I21.9 |
|  |  |  | I21.A1 |
|  |  |  | I21.A9 |
|  |  | 4110 | I24.1 |
|  |  | 4111 | I20.0 |
|  |  | 4118 |  |
|  |  | 41181 | I24.0 |
|  |  | 41189 | I24.8 |
|  |  | 412 | I25.2 |
|  |  | 4130 | I20.8 |
|  |  | 4131 | I20.1 |
|  |  | 4139 | I20.8 |
|  |  |  | I20.9 |
|  |  | 4140 |  |
|  |  | 41400 | I25.10 |
|  |  | 41401 | I25.10 |
|  |  | 41406 | I25.811 |
|  |  | 4142 | I25.82 |
|  |  | 4143 | I25.83 |
|  |  | 4144 | I25.84 |
|  |  | 4148 | I25.5 |
|  |  |  | I25.89 |
|  |  |  | I25.9 |
|  |  | 4149 | I25.9 |
|  |  | V4581 | Z95.1 |
|  |  | V4582 | Z98.61 |
|  | Conduction and Rhythm Disorders | 4260 | I44.2 |
|  |  | 42610 | I44.30 |
|  |  | 42611 | I44.0 |
|  |  | 42612 | I44.1 |
|  |  | 42613 | I44.1 |
|  |  | 4262 | I44.4 |
|  |  |  | I44.5 |
|  |  |  | I44.60 |
|  |  |  | I44.69 |
|  |  | 4263 | I44.7 |
|  |  | 4264 | I45.10 |
|  |  | 42650 | I44.30 |
|  |  |  | I44.39 |
|  |  |  | I45.4 |
|  |  | 42651 | I45.2 |
|  |  | 42652 | I45.2 |
|  |  | 42653 | I45.2 |
|  |  | 42654 | I45.3 |
|  |  | 4266 | I45.5 |
|  |  | 4267 | I45.6 |
|  |  | 42681 | I45.6 |
|  |  | 42682 | I45.81 |
|  |  | 42689 | I45.89 |
|  |  | 4269 | I45.9 |
|  |  | V450 |  |
|  |  | V4500 | Z95.9 |
|  |  | V4501 | Z95.0 |
|  |  | V4502 | Z95.810 |
|  |  | V4509 | Z95.818 |
|  |  | V533 |  |
|  |  | V5331 | Z45.010 |
|  |  |  | Z45.018 |
|  |  | V5332 | Z45.02 |
|  |  | V5339 | Z45.09 |
|  |  | 4270 | I47.1 |
|  |  | 4271 | I47.2 |
|  |  | 4272 | I47.9 |
|  |  | 42731 | I48.91 |
|  |  | 42732 | I48.92 |
|  |  | 42760 | I49.40 |
|  |  | 42761 | I49.1 |
|  |  | 42769 | I49.3 |
|  |  |  | I49.49 |
|  |  | 42781 | I49.5 |
|  |  |  | R00.1 |
|  |  | 42789 | I49.8 |
|  |  |  | R00.1 |
|  |  | 4279 | I49.9 |
|  |  | 7850 | R00.0 |
|  |  | 7851 | R00.2 |
|  |  | 42741 | I49.01 |
|  |  | 42742 | I49.02 |
|  |  | 4275 | I46.9 |
|  | Other Cardiovascular | 3940 | I05.0 |
|  |  | 3941 | I05.1 |
|  |  | 3942 | I05.2 |
|  |  | 3949 | I05.8 |
|  |  | 3950 | I06.0 |
|  |  | 3951 | I06.1 |
|  |  | 3952 | I06.2 |
|  |  | 3959 | I06.8 |
|  |  |  | I06.9 |
|  |  | 3960 | I08.0 |
|  |  | 3961 | I08.0 |
|  |  | 3962 | I08.0 |
|  |  | 3963 | I08.0 |
|  |  | 3968 | I08.8 |
|  |  | 3969 | I08.9 |
|  |  | 3970 | I07.1 |
|  |  |  | I07.2 |
|  |  |  | I07.8 |
|  |  | 3971 | I09.89 |
|  |  | 3979 | I09.1 |
|  |  | 4240 | I34.0 |
|  |  |  | I34.8 |
|  |  | 4241 | I35.0 |
|  |  |  | I35.1 |
|  |  |  | I35.2 |
|  |  |  | I35.8 |
|  |  |  | I35.9 |
|  |  | 4242 | I36.0 |
|  |  |  | I36.8 |
|  |  | 4243 | I37.0 |
|  |  |  | I37.8 |
|  |  | 42490 | I38 |
|  |  | 42491 | I39 |
|  |  | 42499 | I38 |
|  |  | 7852 | R01.1 |
|  |  | 7853 | R01.2 |
|  |  | V422 | Z95.3 |
|  |  | V433 | Z95.2 |
|  |  | 3282 |  |
|  |  | 3640 |  |
|  |  | 3641 |  |
|  |  | 3642 |  |
|  |  | 3643 |  |
|  |  | 7420 | Q01.9 |
|  |  | 7421 | Q02 |
|  |  | 7422 | Q04.1 |
|  |  |  | Q04.2 |
|  |  |  | Q04.3 |
|  |  | 7423 | Q03.0 |
|  |  |  | Q03.1 |
|  |  |  | Q03.8 |
|  |  | 11281 | B37.6 |
|  |  | 11503 | B39.4 |
|  |  |  | I32 |
|  |  | 11504 | B39.4 |
|  |  |  | I39 |
|  |  | 11513 | B39.5 |
|  |  |  | I32 |
|  |  | 11514 | B39.5 |
|  |  |  | I39 |
|  |  | 11593 | B39.9 |
|  |  |  | I32 |
|  |  | 11594 | B39.9 |
|  |  |  | I39 |
|  |  | 1303 | B58.81 |
|  |  | 3910 | I01.0 |
|  |  | 3911 | I01.1 |
|  |  | 3912 | I01.2 |
|  |  | 3918 | I01.8 |
|  |  | 3919 | I01.9 |
|  |  | 3920 | I02.0 |
|  |  | 393 | I09.2 |
|  |  | 3980 | I09.0 |
|  |  | 39890 | I09.9 |
|  |  | 39899 | I09.89 |
|  |  | 4200 | I32 |
|  |  | 42090 | I30.9 |
|  |  | 42091 | I30.0 |
|  |  | 42099 | I30.8 |
|  |  | 4210 | I33.0 |
|  |  | 4211 | I39 |
|  |  | 4219 | I33.9 |
|  |  | 4220 | I41 |
|  |  | 42290 | I40.9 |
|  |  | 42291 | I40.0 |
|  |  |  | I40.1 |
|  |  | 42292 | I40.0 |
|  |  | 42293 | I40.8 |
|  |  | 42299 | I40.8 |
|  |  | 4230 | I31.2 |
|  |  | 4231 | I31.0 |
|  |  | 4232 | I31.1 |
|  |  | 4233 | I31.4 |
|  |  | 4238 | I31.8 |
|  |  | 4239 | I31.9 |
|  |  | 4250 | I42.3 |
|  |  | 4251 |  |
|  |  | 42511 | I42.1 |
|  |  | 42518 | I42.2 |
|  |  | 4252 | I42.8 |
|  |  | 4253 | I42.4 |
|  |  | 4254 | I42.5 |
|  |  |  | I42.8 |
|  |  | 4257 | I43 |
|  |  | 4258 | I43 |
|  |  | 4259 | I42.7 |
|  |  | 4290 | I51.4 |
|  |  | 78650 | R07.9 |
|  |  | 78651 | R07.2 |
|  |  | 78659 | R07.82 |
|  |  |  | R07.89 |
|  |  | 41410 | I25.3 |
|  |  | 41411 | I25.41 |
|  |  | 41412 | I25.42 |
|  |  | 41419 | I25.3 |
|  |  | 4291 | I51.5 |
|  |  | 4292 | I25.10 |
|  |  | 4293 | I51.7 |
|  |  | 4295 | I51.1 |
|  |  | 4296 | I51.2 |
|  |  | 42971 | I51.0 |
|  |  | 42979 | I23.0 |
|  |  | 42981 | I51.89 |
|  |  | 42982 | I51.89 |
|  |  | 42983 | I51.81 |
|  |  | 42989 | I51.3 |
|  |  |  | I51.89 |
|  |  | 4299 | I51.9 |
|  |  | 34660 | G43.609 |
|  |  | 34661 | G43.619 |
|  |  | 34662 | G43.601 |
|  |  | 34663 | G43.611 |
|  |  | 430 | I60.9 |
|  |  | 431 | I61.9 |
|  |  | 4320 | I62.1 |
|  |  | 4321 | I62.00 |
|  |  | 4329 | I62.9 |
|  |  | 43301 | I63.22 |
|  |  | 43311 | I63.139 |
|  |  |  | I63.239 |
|  |  | 43321 | I63.019 |
|  |  |  | I63.119 |
|  |  |  | I63.219 |
|  |  | 43331 | I63.59 |
|  |  | 43381 | I63.59 |
|  |  | 43391 | I63.20 |
|  |  | 4340 |  |
|  |  | 43400 | I66.09 |
|  |  |  | I66.19 |
|  |  |  | I66.29 |
|  |  | 43401 | I63.30 |
|  |  | 4341 |  |
|  |  | 43410 | I66.09 |
|  |  |  | I66.19 |
|  |  |  | I66.29 |
|  |  |  | I66.9 |
|  |  | 43411 | I63.40 |
|  |  | 4349 |  |
|  |  | 43490 | I66.9 |
|  |  | 43491 | I63.50 |
|  |  | 436 | I67.89 |
|  |  | 4330 |  |
|  |  | 43300 | I65.1 |
|  |  | 4331 |  |
|  |  | 43310 | I65.29 |
|  |  | 4332 |  |
|  |  | 43320 | I65.09 |
|  |  | 4333 |  |
|  |  | 43330 | I65.8 |
|  |  | 4338 |  |
|  |  | 43380 | I65.8 |
|  |  | 4339 |  |
|  |  | 43390 | I65.9 |
|  |  | 4370 | I67.2 |
|  |  | 4371 | I67.81 |
|  |  |  | I67.82 |
|  |  |  | I67.89 |
|  |  | 4373 | I67.1 |
|  |  | 4374 | I67.7 |
|  |  | 4375 | I67.5 |
|  |  | 4376 | I67.6 |
|  |  | 4377 | G45.4 |
|  |  | 4378 | I67.89 |
|  |  | 4379 | I67.9 |
|  |  | 4350 | G45.0 |
|  |  | 4351 | G45.0 |
|  |  | 4352 | G45.8 |
|  |  | 4353 | G45.0 |
|  |  | 4358 | G45.1 |
|  |  |  | G45.8 |
|  |  | 4359 | G45.9 |
|  |  |  | I67.848 |
|  |  | 4380 | I69.910 |
|  |  |  | I69.911 |
|  |  |  | I69.912 |
|  |  |  | I69.913 |
|  |  |  | I69.914 |
|  |  |  | I69.915 |
|  |  |  | I69.918 |
|  |  |  | I69.919 |
|  |  | 43810 | I69.928 |
|  |  | 43811 | I69.920 |
|  |  | 43812 | I69.921 |
|  |  | 43813 | I69.922 |
|  |  | 43814 | I69.923 |
|  |  | 43819 | I69.928 |
|  |  | 43820 | I69.959 |
|  |  | 43821 | I69.951 |
|  |  |  | I69.952 |
|  |  | 43822 | I69.953 |
|  |  |  | I69.954 |
|  |  | 43830 | I69.939 |
|  |  | 43831 | I69.931 |
|  |  |  | I69.932 |
|  |  | 43832 | I69.933 |
|  |  |  | I69.934 |
|  |  | 43840 | I69.949 |
|  |  | 43841 | I69.941 |
|  |  |  | I69.942 |
|  |  | 43842 | I69.943 |
|  |  |  | I69.944 |
|  |  | 43850 | I69.969 |
|  |  | 43851 | I69.961 |
|  |  |  | I69.962 |
|  |  | 43852 | I69.963 |
|  |  |  | I69.964 |
|  |  | 43853 | I69.965 |
|  |  | 4386 | I69.998 |
|  |  | 4387 | I69.998 |
|  |  | 43881 | I69.990 |
|  |  | 43882 | I69.991 |
|  |  | 43883 | I69.992 |
|  |  | 43884 | I69.993 |
|  |  | 43885 | I69.998 |
|  |  | 43889 | I69.898 |
|  |  |  | I69.998 |
|  |  | 4389 | I69.90 |
|  |  | 4400 | I70.0 |
|  |  | 4401 | I70.1 |
|  |  | 4402 |  |
|  |  | 44020 | I70.209 |
|  |  | 44021 | I70.219 |
|  |  | 44022 | I70.229 |
|  |  | 44023 | I70.25 |
|  |  | 44029 | I70.299 |
|  |  | 4404 | I70.92 |
|  |  | 4408 | I70.8 |
|  |  | 4409 | I70.90 |
|  |  |  | I70.91 |
|  |  | 4439 | I73.9 |
|  |  | 5570 | K55.011 |
|  |  |  | K55.012 |
|  |  |  | K55.019 |
|  |  |  | K55.021 |
|  |  |  | K55.022 |
|  |  |  | K55.029 |
|  |  |  | K55.031 |
|  |  |  | K55.032 |
|  |  |  | K55.039 |
|  |  |  | K55.041 |
|  |  |  | K55.042 |
|  |  |  | K55.049 |
|  |  |  | K55.051 |
|  |  |  | K55.052 |
|  |  |  | K55.059 |
|  |  |  | K55.069 |
|  |  |  | K55.061 |
|  |  |  | K55.062 |
|  |  |  | K55.31 |
|  |  |  | K55.32 |
|  |  |  | K55.33 |
|  |  | 5571 | K55.1 |
|  |  | 5579 | K55.9 |
|  |  | 4410 |  |
|  |  | 44100 | I71.00 |
|  |  | 44101 | I71.01 |
|  |  | 44102 | I71.02 |
|  |  | 44103 | I71.03 |
|  |  | 4411 | I71.1 |
|  |  | 4412 | I71.2 |
|  |  | 4413 | I71.3 |
|  |  | 4414 | I71.4 |
|  |  | 4415 | I71.8 |
|  |  | 4416 | I71.5 |
|  |  | 4417 | I71.6 |
|  |  | 4419 | I71.9 |
|  |  | 4420 | I72.1 |
|  |  | 4421 | I72.2 |
|  |  | 4422 | I72.3 |
|  |  | 4423 | I72.4 |
|  |  | 44281 | I72.0 |
|  |  |  | I72.5 |
|  |  |  | I72.6 |
|  |  | 44282 | I72.8 |
|  |  | 44283 | I72.8 |
|  |  | 44284 | I72.8 |
|  |  | 44289 | I72.8 |
|  |  | 4429 | I72.9 |
|  |  | 44321 | I77.71 |
|  |  | 44322 | I77.72 |
|  |  | 44323 | I77.73 |
|  |  | 44324 | I77.74 |
|  |  | 44329 | I77.75 |
|  |  |  | I77.76 |
|  |  |  | I77.77 |
|  |  |  | I77.79 |
|  |  | 44770 | I77.819 |
|  |  | 44771 | I77.810 |
|  |  | 44772 | I77.811 |
|  |  | 44773 | I77.812 |
|  |  | 4440 |  |
|  |  | 44401 | I74.01 |
|  |  | 44409 | I74.09 |
|  |  | 4441 | I74.11 |
|  |  | 44421 | I74.2 |
|  |  | 44422 | I74.3 |
|  |  | 44481 | I74.5 |
|  |  | 44489 | I74.8 |
|  |  | 4449 | I74.9 |
|  |  | 44501 | I75.019 |
|  |  | 44502 | I75.029 |
|  |  | 44581 | I75.81 |
|  |  | 44589 | I75.89 |
|  |  | 4430 | I73.00 |
|  |  | 4431 | I73.1 |
|  |  | 44381 | I79.8 |
|  |  | 44382 | I73.81 |
|  |  | 44389 | I73.89 |
|  |  | 4460 | M30.0 |
|  |  | 4461 | M30.3 |
|  |  | 4462 |  |
|  |  | 44620 | M31.0 |
|  |  | 44621 | M31.0 |
|  |  | 44629 | M31.0 |
|  |  | 4463 | M31.2 |
|  |  | 4464 | M31.30 |
|  |  | 4465 | M31.6 |
|  |  | 4466 | M31.1 |
|  |  | 4467 | M31.4 |
|  |  | 4470 | I77.0 |
|  |  | 4471 | I77.1 |
|  |  | 4472 | I77.2 |
|  |  | 4473 | I77.3 |
|  |  | 4474 | I77.4 |
|  |  | 4475 | I77.5 |
|  |  | 4476 | I77.6 |
|  |  | 4478 | I77.3 |
|  |  |  | I77.89 |
|  |  | 4479 | I77.9 |
|  |  | 4480 | I78.0 |
|  |  | 4481 | I78.1 |
|  |  | 4489 | I78.8 |
|  |  |  | I78.9 |
|  |  | 4580 | I95.1 |
|  |  | 4581 | I95.89 |
|  |  | 4588 | I95.89 |
|  |  | 4589 | I95.9 |
|  |  | 4590 | R58 |
|  |  | 45989 | I99.8 |
|  |  | 4599 | I87.9 |
|  |  |  | I99.9 |
|  |  | 7859 | R09.89 |
|  |  | 79430 | R94.30 |
|  |  | 79431 | R94.31 |
|  |  | 79439 | R94.39 |
|  |  | 7962 | R03.0 |
|  |  | V125 |  |
|  |  | V1250 | Z86.79 |
|  |  | V1253 | Z86.74 |
|  |  | V1254 | Z86.73 |
|  |  | V1259 | Z86.79 |
|  |  | V151 | Z98.890 |
|  |  | V421 | Z94.1 |
|  |  | V432 |  |
|  |  | V4321 | Z95.811 |
|  |  | V4322 | Z95.812 |
|  |  | V434 | Z95.828 |
|  |  | V717 | Z03.89 |
|  | Birth Defects (Non-CHD) | 7500 | Q38.1 |
|  |  | 75010 | Q38.3 |
|  |  | 75011 | Q38.3 |
|  |  | 75012 | Q38.3 |
|  |  | 75013 | Q38.3 |
|  |  | 75015 | Q38.2 |
|  |  | 75016 | Q38.3 |
|  |  | 75019 | Q38.3 |
|  |  | 75021 | Q38.4 |
|  |  | 75022 | Q38.4 |
|  |  | 75023 | Q38.4 |
|  |  | 75024 | Q38.4 |
|  |  | 75025 | Q38.0 |
|  |  | 75026 | Q38.6 |
|  |  | 75027 | Q38.7 |
|  |  | 75029 | Q38.8 |
|  |  | 7503 | Q39.0 |
|  |  |  | Q39.1 |
|  |  |  | Q39.2 |
|  |  |  | Q39.3 |
|  |  |  | Q39.4 |
|  |  | 7504 | Q39.5 |
|  |  |  | Q39.6 |
|  |  |  | Q39.8 |
|  |  | 7505 | Q40.0 |
|  |  | 7506 | Q40.1 |
|  |  | 7507 | Q39.5 |
|  |  |  | Q40.2 |
|  |  | 7508 | Q40.8 |
|  |  | 7509 | Q38.5 |
|  |  |  | Q40.9 |
|  |  | 7510 | Q43.0 |
|  |  | 7511 | Q41.9 |
|  |  | 7512 | Q42.9 |
|  |  | 7513 | Q43.1 |
|  |  | 7514 | Q43.3 |
|  |  | 7515 | Q43.4 |
|  |  |  | Q43.5 |
|  |  |  | Q43.7 |
|  |  |  | Q43.8 |
|  |  | 75160 | Q44.1 |
|  |  |  | Q44.5 |
|  |  |  | Q44.7 |
|  |  | 75161 | Q44.2 |
|  |  |  | Q44.3 |
|  |  | 75162 | Q44.6 |
|  |  | 75169 | Q44.1 |
|  |  |  | Q44.4 |
|  |  |  | Q44.5 |
|  |  |  | Q44.7 |
|  |  | 7517 | Q45.0 |
|  |  |  | Q45.1 |
|  |  |  | Q45.3 |
|  |  | 7518 | Q45.8 |
|  |  | 7519 | Q45.9 |
|  |  | V1367 | Z87.738 |
|  |  | 7520 | Q50.01 |
|  |  |  | Q50.02 |
|  |  |  | Q50.31 |
|  |  |  | Q50.32 |
|  |  |  | Q50.39 |
|  |  | 75210 | Q50.6 |
|  |  | 75211 | Q50.4 |
|  |  |  | Q50.5 |
|  |  | 75219 | Q50.6 |
|  |  | 7522 | Q51.10 |
|  |  |  | Q51.2 |
|  |  | 7523 |  |
|  |  | 75231 | Q51.0 |
|  |  | 75232 | Q51.811 |
|  |  | 75233 | Q51.4 |
|  |  | 75234 | Q51.3 |
|  |  | 75235 | Q51.2 |
|  |  | 75236 | Q51.810 |
|  |  | 75239 | Q51.818 |
|  |  | 75240 | Q52.9 |
|  |  | 75241 | Q51.6 |
|  |  | 75242 | Q52.4 |
|  |  | 75243 | Q51.5 |
|  |  | 75244 | Q51.820 |
|  |  | 75245 | Q52.0 |
|  |  | 75246 | Q52.11 |
|  |  | 75247 | Q52.120 |
|  |  |  | Q52.121 |
|  |  |  | Q52.122 |
|  |  |  | Q52.123 |
|  |  |  | Q52.124 |
|  |  |  | Q52.129 |
|  |  | 75249 | Q51.0 |
|  |  |  | Q51.5 |
|  |  |  | Q51.821 |
|  |  |  | Q51.828 |
|  |  |  | Q52.10 |
|  |  |  | Q52.2 |
|  |  |  | Q52.4 |
|  |  |  | Q52.5 |
|  |  |  | Q52.6 |
|  |  |  | Q52.70 |
|  |  |  | Q52.71 |
|  |  |  | Q52.79 |
|  |  |  | Q52.8 |
|  |  | 7525 |  |
|  |  | 75251 | Q53.13 |
|  |  |  | Q53.9 |
|  |  | 75252 | Q55.22 |
|  |  | 7526 |  |
|  |  | 75261 | Q54.9 |
|  |  | 75262 | Q64.0 |
|  |  | 75263 | Q54.4 |
|  |  | 75264 | Q55.62 |
|  |  | 75265 | Q55.64 |
|  |  | 75269 | Q55.63 |
|  |  |  | Q55.69 |
|  |  | 7527 | Q56.3 |
|  |  |  | Q56.4 |
|  |  | 7528 |  |
|  |  | 75281 | Q55.23 |
|  |  | 75289 | Q52.8 |
|  |  |  | Q55.0 |
|  |  |  | Q55.1 |
|  |  |  | Q55.21 |
|  |  |  | Q55.29 |
|  |  |  | Q55.3 |
|  |  |  | Q55.8 |
|  |  | 7529 | Q52.9 |
|  |  |  | Q55.9 |
|  |  | 7530 | Q60.2 |
|  |  |  | Q60.5 |
|  |  | 7531 |  |
|  |  | 75310 | Q61.00 |
|  |  |  | Q61.9 |
|  |  | 75311 | Q61.01 |
|  |  | 75312 | Q61.3 |
|  |  | 75313 | Q61.2 |
|  |  | 75314 | Q61.19 |
|  |  | 75315 | Q61.4 |
|  |  | 75316 | Q61.5 |
|  |  | 75317 | Q61.5 |
|  |  | 75319 | Q61.02 |
|  |  |  | Q61.8 |
|  |  | 7532 |  |
|  |  | 75320 | Q62.39 |
|  |  | 75321 | Q62.11 |
|  |  | 75322 | Q62.12 |
|  |  | 75323 | Q62.31 |
|  |  | 75329 | Q62.10 |
|  |  |  | Q62.11 |
|  |  | 7533 | Q63.0 |
|  |  |  | Q63.1 |
|  |  |  | Q63.2 |
|  |  |  | Q63.3 |
|  |  |  | Q63.8 |
|  |  | 7534 | Q62.4 |
|  |  |  | Q62.5 |
|  |  |  | Q62.21 |
|  |  |  | Q62.22 |
|  |  |  | Q62.23 |
|  |  |  | Q62.8 |
|  |  | 7535 | Q64.10 |
|  |  |  | Q64.19 |
|  |  | 7536 | Q64.2 |
|  |  |  | Q64.31 |
|  |  |  | Q64.32 |
|  |  |  | Q64.33 |
|  |  |  | Q64.39 |
|  |  | 7537 | Q64.4 |
|  |  | 7538 | Q64.5 |
|  |  |  | Q64.6 |
|  |  |  | Q64.71 |
|  |  |  | Q64.73 |
|  |  |  | Q64.74 |
|  |  |  | Q64.75 |
|  |  |  | Q64.79 |
|  |  | 7539 | Q64.9 |
|  |  | V1361 | Z87.710 |
|  |  | V1362 | Z87.718 |
|  |  | 7400 | Q00.0 |
|  |  | 7401 | Q00.1 |
|  |  | 7402 | Q00.2 |
|  |  | 74100 | Q05.4 |
|  |  |  | Q07.02 |
|  |  |  | Q07.03 |
|  |  | 74101 | Q05.0 |
|  |  | 74102 | Q05.1 |
|  |  | 74103 | Q05.2 |
|  |  | 74190 | Q05.8 |
|  |  |  | Q07.01 |
|  |  | 74191 | Q05.5 |
|  |  | 74192 | Q05.6 |
|  |  | 74193 | Q05.7 |
|  |  | 7420 | Q01.9 |
|  |  | 7421 | Q02 |
|  |  | 7422 | Q04.1 |
|  |  |  | Q04.2 |
|  |  |  | Q04.3 |
|  |  | 7423 | Q03.0 |
|  |  |  | Q03.1 |
|  |  |  | Q03.8 |
|  |  | 7424 | Q04.5 |
|  |  |  | Q04.6 |
|  |  |  | Q04.8 |
|  |  | 74251 | Q06.2 |
|  |  | 74253 | Q06.4 |
|  |  | 74259 | Q06.0 |
|  |  |  | Q06.1 |
|  |  |  | Q06.3 |
|  |  |  | Q06.8 |
|  |  | 7428 | G90.1 |
|  |  |  | Q07.8 |
|  |  | 7429 | Q07.9 |
|  |  | V1363 | Z87.728 |
|  |  | 74300 | Q11.1 |
|  |  | 74303 | Q11.0 |
|  |  | 74306 | Q11.2 |
|  |  | 74310 | Q11.2 |
|  |  | 74311 | Q11.2 |
|  |  | 74312 | Q11.2 |
|  |  | 74320 | Q15.0 |
|  |  | 74321 | Q15.0 |
|  |  | 74322 | Q15.0 |
|  |  | 74330 | Q12.0 |
|  |  | 74331 | Q12.0 |
|  |  | 74332 | Q12.0 |
|  |  | 74333 | Q12.0 |
|  |  | 74334 | Q12.0 |
|  |  | 74335 | Q12.3 |
|  |  | 74336 | Q12.4 |
|  |  |  | Q12.8 |
|  |  | 74337 | Q12.1 |
|  |  | 74339 | Q12.9 |
|  |  | 74341 | Q13.4 |
|  |  | 74342 | Q13.3 |
|  |  | 74343 | Q13.3 |
|  |  | 74344 | Q13.4 |
|  |  |  | Q13.81 |
|  |  |  | Q15.0 |
|  |  | 74345 | Q13.1 |
|  |  | 74346 | Q13.0 |
|  |  |  | Q13.2 |
|  |  | 74347 | Q13.5 |
|  |  | 74348 | Q13.89 |
|  |  | 74349 | Q13.89 |
|  |  | 74351 | Q14.0 |
|  |  | 74352 | Q14.8 |
|  |  | 74353 | Q14.3 |
|  |  | 74354 | Q14.8 |
|  |  | 74355 | Q14.8 |
|  |  | 74356 | Q14.1 |
|  |  | 74357 | Q14.2 |
|  |  | 74358 | Q14.8 |
|  |  | 74359 | Q14.8 |
|  |  | 74361 | Q10.0 |
|  |  | 74362 | Q10.1 |
|  |  |  | Q10.2 |
|  |  |  | Q10.3 |
|  |  | 74363 | Q10.3 |
|  |  | 74364 | Q10.6 |
|  |  | 74365 | Q10.4 |
|  |  |  | Q10.6 |
|  |  | 74366 | Q10.7 |
|  |  | 74369 | Q10.3 |
|  |  |  | Q10.6 |
|  |  |  | Q10.7 |
|  |  | 7438 | Q11.3 |
|  |  |  | Q15.8 |
|  |  | 7439 | Q15.9 |
|  |  | 74400 | Q16.9 |
|  |  | 74401 | Q16.0 |
|  |  | 74402 | Q16.1 |
|  |  | 74403 | Q16.4 |
|  |  | 74404 | Q16.3 |
|  |  | 74405 | Q16.5 |
|  |  | 74409 | Q16.9 |
|  |  | 7441 | Q17.0 |
|  |  | 74421 | Q17.8 |
|  |  | 74422 | Q17.1 |
|  |  | 74423 | Q17.2 |
|  |  | 74424 | Q16.2 |
|  |  | 74429 | Q17.3 |
|  |  |  | Q17.4 |
|  |  |  | Q17.5 |
|  |  |  | Q17.8 |
|  |  | 7443 | Q17.9 |
|  |  | 74441 | Q18.0 |
|  |  | 74442 | Q18.0 |
|  |  | 74443 | Q18.2 |
|  |  | 74446 | Q18.1 |
|  |  | 74447 | Q18.1 |
|  |  | 74449 | Q18.2 |
|  |  | 7445 | Q18.3 |
|  |  | 74481 | Q18.6 |
|  |  | 74482 | Q18.7 |
|  |  | 74483 | Q18.5 |
|  |  | 74484 | Q18.5 |
|  |  | 74489 | Q18.8 |
|  |  | 7449 | Q18.9 |
|  |  | 7480 | Q30.0 |
|  |  | 7481 | Q30.1 |
|  |  |  | Q30.2 |
|  |  |  | Q30.8 |
|  |  | 7482 | Q31.0 |
|  |  | 7483 | Q31.1 |
|  |  |  | Q31.3 |
|  |  |  | Q31.8 |
|  |  |  | Q32.1 |
|  |  |  | Q32.4 |
|  |  | 7484 | Q33.0 |
|  |  | 7485 | Q33.2 |
|  |  |  | Q33.3 |
|  |  |  | Q33.6 |
|  |  | 74860 | Q33.9 |
|  |  | 74861 | Q33.4 |
|  |  | 74869 | Q33.1 |
|  |  |  | Q33.5 |
|  |  |  | Q33.8 |
|  |  | 7488 | Q34.0 |
|  |  |  | Q34.1 |
|  |  |  | Q34.8 |
|  |  | 7489 | Q34.9 |
|  |  | 74900 | Q35.9 |
|  |  | 74901 | Q35.9 |
|  |  | 74902 | Q35.7 |
|  |  |  | Q35.9 |
|  |  | 74903 | Q35.9 |
|  |  | 74904 | Q35.9 |
|  |  | 74910 | Q36.9 |
|  |  | 74911 | Q36.9 |
|  |  | 74912 | Q36.9 |
|  |  | 74913 | Q36.0 |
|  |  | 74914 | Q36.0 |
|  |  | 74920 | Q37.9 |
|  |  | 74921 | Q37.9 |
|  |  | 74922 | Q37.9 |
|  |  | 74923 | Q37.8 |
|  |  | 74924 | Q37.8 |
|  |  | 74925 | Q37.9 |
|  |  | 7540 | Q67.0 |
|  |  |  | Q67.1 |
|  |  |  | Q67.2 |
|  |  |  | Q67.3 |
|  |  |  | Q67.4 |
|  |  | 7541 | Q68.0 |
|  |  | 7542 | Q67.5 |
|  |  |  | Q76.3 |
|  |  |  | Q76.425 |
|  |  |  | Q76.426 |
|  |  |  | Q76.427 |
|  |  |  | Q76.428 |
|  |  | 75430 | Q65.00 |
|  |  | 75431 | Q65.1 |
|  |  | 75432 | Q65.30 |
|  |  | 75433 | Q65.4 |
|  |  | 75435 | Q65.01 |
|  |  |  | Q65.32 |
|  |  |  | Q65.02 |
|  |  |  | Q65.31 |
|  |  | 75440 | Q68.2 |
|  |  | 75441 | Q68.2 |
|  |  | 75442 | Q68.3 |
|  |  | 75443 | Q68.4 |
|  |  | 75444 | Q68.5 |
|  |  | 75450 | Q66.0 |
|  |  | 75451 | Q66.1 |
|  |  | 75452 | Q66.2 |
|  |  | 75453 | Q66.3 |
|  |  | 75459 | Q66.1 |
|  |  |  | Q66.3 |
|  |  | 75460 | Q66.4 |
|  |  | 75461 | Q66.50 |
|  |  |  | Q66.80 |
|  |  | 75462 | Q66.4 |
|  |  | 75469 | Q66.6 |
|  |  | 75470 | Q66.89 |
|  |  | 75471 | Q66.7 |
|  |  | 75479 | Q66.89 |
|  |  | 75481 | Q67.6 |
|  |  | 75482 | Q67.7 |
|  |  | 75489 | Q67.8 |
|  |  |  | Q68.1 |
|  |  |  | Q74.3 |
|  |  | 75500 | Q69.9 |
|  |  | 75501 | Q69.0 |
|  |  |  | Q69.1 |
|  |  | 75502 | Q69.2 |
|  |  | 75510 | Q70.4 |
|  |  |  | Q70.9 |
|  |  | 75511 | Q70.10 |
|  |  | 75512 | Q70.00 |
|  |  | 75513 | Q70.30 |
|  |  | 75514 | Q70.20 |
|  |  | 75520 | Q71.889 |
|  |  |  | Q71.90 |
|  |  | 75521 | Q71.00 |
|  |  | 75522 | Q71.00 |
|  |  | 75523 | Q71.10 |
|  |  | 75524 | Q71.10 |
|  |  | 75525 | Q71.20 |
|  |  | 75526 | Q71.40 |
|  |  | 75527 | Q71.50 |
|  |  | 75528 | Q71.30 |
|  |  | 75529 | Q71.30 |
|  |  | 75530 | Q72.899 |
|  |  | 75531 | Q72.00 |
|  |  | 75532 | Q72.899 |
|  |  | 75533 | Q72.10 |
|  |  | 75534 | Q72.40 |
|  |  | 75535 | Q72.20 |
|  |  | 75536 | Q72.50 |
|  |  | 75537 | Q72.60 |
|  |  | 75538 | Q72.30 |
|  |  |  | Q72.70 |
|  |  | 75539 | Q72.30 |
|  |  |  | Q72.70 |
|  |  | 7554 | Q73.0 |
|  |  |  | Q73.1 |
|  |  |  | Q73.8 |
|  |  | 75550 | Q74.9 |
|  |  | 75551 | Q68.8 |
|  |  | 75552 | Q68.8 |
|  |  | 75553 | Q74.0 |
|  |  | 75554 | Q74.0 |
|  |  | 75555 | Q87.0 |
|  |  | 75556 | Q74.0 |
|  |  | 75557 | Q74.0 |
|  |  | 75558 | Q71.60 |
|  |  | 75559 | Q74.0 |
|  |  | 75560 | Q74.2 |
|  |  | 75561 | Q65.81 |
|  |  | 75562 | Q65.82 |
|  |  | 75563 | Q65.89 |
|  |  | 75564 | Q68.2 |
|  |  |  | Q74.1 |
|  |  | 75565 | Q74.2 |
|  |  | 75566 | Q65.89 |
|  |  | 75567 | Q65.89 |
|  |  | 75569 | Q74.2 |
|  |  | 7558 | Q74.8 |
|  |  | 7559 | Q74.9 |
|  |  | 7560 | Q75.0 |
|  |  |  | Q75.2 |
|  |  |  | Q75.9 |
|  |  | 75610 | Q76.49 |
|  |  | 75611 | Q76.2 |
|  |  | 75612 | Q76.2 |
|  |  | 75613 | Q76.49 |
|  |  | 75614 | Q76.49 |
|  |  | 75615 | Q76.49 |
|  |  | 75616 | Q76.1 |
|  |  | 75617 | Q76.0 |
|  |  | 75619 | Q76.419 |
|  |  |  | Q76.49 |
|  |  | 7562 | Q76.5 |
|  |  | 7563 | Q76.6 |
|  |  |  | Q76.7 |
|  |  |  | Q77.2 |
|  |  | 7564 | Q77.1 |
|  |  |  | Q77.4 |
|  |  |  | Q77.8 |
|  |  |  | Q78.4 |
|  |  | 75650 | Q78.9 |
|  |  | 75651 | Q78.0 |
|  |  | 75652 | Q78.2 |
|  |  | 75653 | Q78.8 |
|  |  | 75654 | Q78.1 |
|  |  | 75655 | Q77.6 |
|  |  | 75656 | Q78.3 |
|  |  | 75659 | Q78.8 |
|  |  | 7566 | Q79.0 |
|  |  |  | Q79.1 |
|  |  | 7567 |  |
|  |  | 75670 | Q79.59 |
|  |  | 75671 | Q79.4 |
|  |  |  | Q79.51 |
|  |  | 75672 | Q79.2 |
|  |  | 75673 | Q79.3 |
|  |  | 75679 | Q79.59 |
|  |  | 75681 | Q79.8 |
|  |  | 75682 | Q79.8 |
|  |  | 75683 | Q79.6 |
|  |  | 75689 | Q79.8 |
|  |  | 7569 | Q68.8 |
|  |  |  | Q79.8 |
|  |  |  | Q79.9 |
|  |  | 7570 | Q82.0 |
|  |  | 7571 | Q80.3 |
|  |  |  | Q80.4 |
|  |  |  | Q80.9 |
|  |  | 7572 | Q82.8 |
|  |  | 75731 | Q82.4 |
|  |  | 75732 | Q82.5 |
|  |  | 75733 | Q82.1 |
|  |  |  | Q82.2 |
|  |  | 75739 | Q81.9 |
|  |  |  | Q82.6 |
|  |  |  | Q82.8 |
|  |  | 7574 | Q84.0 |
|  |  |  | Q84.1 |
|  |  |  | Q84.2 |
|  |  | 7575 | Q84.3 |
|  |  |  | Q84.4 |
|  |  |  | Q84.5 |
|  |  |  | Q84.6 |
|  |  | 7576 | Q83.0 |
|  |  |  | Q83.1 |
|  |  |  | Q83.2 |
|  |  |  | Q83.3 |
|  |  |  | Q83.8 |
|  |  | 7578 | Q84.8 |
|  |  | 7579 | Q84.9 |
|  |  | 7580 | Q90.9 |
|  |  | 7581 | Q91.7 |
|  |  | 7582 | Q91.3 |
|  |  | 7583 |  |
|  |  | 75831 | Q93.4 |
|  |  | 75832 | Q93.81 |
|  |  | 75833 | Q93.88 |
|  |  | 75839 | Q93.3 |
|  |  |  | Q93.7 |
|  |  |  | Q93.89 |
|  |  | 7584 | Q95.0 |
|  |  | 7585 | Q92.8 |
|  |  | 7586 | Q96.9 |
|  |  | 7587 | Q98.4 |
|  |  | 75881 | Q97.0 |
|  |  |  | Q97.1 |
|  |  |  | Q97.2 |
|  |  |  | Q97.8 |
|  |  |  | Q98.5 |
|  |  |  | Q98.7 |
|  |  |  | Q98.8 |
|  |  |  | Q99.8 |
|  |  | 7588 |  |
|  |  | 75889 | Q99.8 |
|  |  | 7589 | Q99.9 |
|  |  | 7590 | Q89.01 |
|  |  |  | Q89.09 |
|  |  | 7591 | Q89.1 |
|  |  | 7592 | Q89.2 |
|  |  | 7593 | Q89.3 |
|  |  | 7594 | Q89.4 |
|  |  | 7595 | Q85.1 |
|  |  | 7596 | Q85.8 |
|  |  | 7597 | Q89.7 |
|  |  | 7598 |  |
|  |  | 75981 | Q87.1 |
|  |  | 75982 | Q87.40 |
|  |  | 75983 | Q99.2 |
|  |  | 75989 | E78.71 |
|  |  |  | E78.72 |
|  |  |  | Q87.2 |
|  |  |  | Q87.3 |
|  |  |  | Q87.5 |
|  |  |  | Q87.81 |
|  |  |  | Q87.82 |
|  |  |  | Q87.89 |
|  |  | 7599 | Q89.9 |
|  |  | 7952 | R89.8 |
|  |  | V136 |  |
|  |  | V1364 | Z87.720 |
|  |  |  | Z87.721 |
|  |  |  | Z87.730 |
|  |  |  | Z87.790 |
|  |  | V1366 | Z87.75 |
|  |  | V1368 | Z87.76 |
|  |  | V1369 | Z87.798 |
|  | Diabetes Mellitus | CM_DM, CM_DMCX |  |
|  | Hyperlipidemia | 2720 | E78.00 |
|  |  |  | E78.01 |
|  |  | 2721 | E78.1 |
|  |  | 2722 | E78.2 |
|  |  | 2723 | E78.3 |
|  |  | 2724 | E78.4 |
|  |  |  | E78.5 |
|  | Mental Health | 3090 | F43.21 |
|  |  | 3091 | F43.21 |
|  |  | 30922 | F94.8 |
|  |  | 30923 | F94.8 |
|  |  | 30924 | F43.22 |
|  |  | 30928 | F43.23 |
|  |  | 30929 | F43.29 |
|  |  |  | F94.8 |
|  |  | 3093 | F43.24 |
|  |  | 3094 | F43.25 |
|  |  | 30982 | F43.8 |
|  |  | 30983 | F43.8 |
|  |  | 30989 | F43.8 |
|  |  | 3099 | F43.20 |
|  |  | 29384 | F06.4 |
|  |  | 30000 | F41.9 |
|  |  | 30001 | F41.0 |
|  |  | 30002 | F41.1 |
|  |  | 30009 | F41.8 |
|  |  | 30010 | F44.9 |
|  |  | 30020 | F40.9 |
|  |  | 30021 | F40.01 |
|  |  | 30022 | F40.02 |
|  |  | 30023 | F40.10 |
|  |  | 30029 | F40.218 |
|  |  |  | F40.240 |
|  |  |  | F40.241 |
|  |  |  | F40.8 |
|  |  | 3003 | F42.2 |
|  |  |  | F42.3 |
|  |  |  | F42.8 |
|  |  |  | F42.9 |
|  |  | 3005 | F48.8 |
|  |  | 30089 | F45.8 |
|  |  |  | F48.8 |
|  |  | 3009 | F48.9 |
|  |  |  | F99 |
|  |  | 3080 | F43.0 |
|  |  | 3081 | F43.0 |
|  |  | 3082 | F43.0 |
|  |  | 3083 | F43.0 |
|  |  | 3084 | F43.0 |
|  |  | 3089 | F43.0 |
|  |  |  | R45.7 |
|  |  | 30981 | F43.10 |
|  |  |  | F43.12 |
|  |  | 3130 | F93.8 |
|  |  | 3131 | F93.8 |
|  |  | 31321 | F93.8 |
|  |  | 31322 | F93.8 |
|  |  | 3133 | F93.8 |
|  |  | 31382 | F93.8 |
|  |  | 31383 | F93.8 |
|  |  | 31200 | F91.1 |
|  |  | 31201 | F91.1 |
|  |  | 31202 | F91.1 |
|  |  | 31203 | F91.1 |
|  |  | 31210 | F91.8 |
|  |  | 31211 | F91.8 |
|  |  | 31212 | F91.8 |
|  |  | 31213 | F91.8 |
|  |  | 31220 | F91.2 |
|  |  | 31221 | F91.2 |
|  |  | 31222 | F91.2 |
|  |  | 31223 | F91.2 |
|  |  | 3124 | F91.8 |
|  |  | 3128 |  |
|  |  | 31281 | F91.1 |
|  |  | 31282 | F91.2 |
|  |  | 31289 | F91.8 |
|  |  | 3129 | F91.9 |
|  |  | 31381 | F91.3 |
|  |  | 31400 | F90.0 |
|  |  |  | F90.9 |
|  |  | 31401 | F90.1 |
|  |  |  | F90.2 |
|  |  |  | F90.9 |
|  |  | 3141 | F90.8 |
|  |  | 3142 | F90.8 |
|  |  | 3148 | F90.8 |
|  |  | 3149 | F90.9 |
|  |  | 2900 | F03.90 |
|  |  | 29010 | F03.90 |
|  |  | 29011 | F03.90 |
|  |  | 29012 | F03.90 |
|  |  |  | F05 |
|  |  | 29013 | F03.90 |
|  |  | 29020 | F03.90 |
|  |  |  | F05 |
|  |  | 29021 | F03.90 |
|  |  | 2903 | F03.90 |
|  |  |  | F05 |
|  |  | 29040 | F01.50 |
|  |  | 29041 | F01.51 |
|  |  | 29042 | F01.51 |
|  |  | 29043 | F01.51 |
|  |  | 2908 | F03.90 |
|  |  | 2909 | F03.90 |
|  |  | 2930 | F05 |
|  |  | 2931 | F05 |
|  |  | 2940 | F04 |
|  |  | 2941 |  |
|  |  | 29410 | F02.80 |
|  |  | 29411 | F02.81 |
|  |  | 29420 | F03.90 |
|  |  | 29421 | F03.91 |
|  |  | 2948 | F06.0 |
|  |  |  | F06.8 |
|  |  | 2949 | F06.8 |
|  |  | 3100 | F07.0 |
|  |  | 3102 | F07.81 |
|  |  | 3108 |  |
|  |  | 31081 | F48.2 |
|  |  | 31089 | F07.89 |
|  |  | 3109 | F09 |
|  |  | 3310 | G30.9 |
|  |  | 3311 |  |
|  |  | 33111 | G31.01 |
|  |  | 33119 | G31.09 |
|  |  | 3312 | G31.1 |
|  |  | 33182 | G31.83 |
|  |  | 797 | R41.81 |
|  |  | 29900 | F84.0 |
|  |  | 29901 | F84.0 |
|  |  | 29910 | F84.3 |
|  |  | 29911 | F84.3 |
|  |  | 29980 | F84.5 |
|  |  |  | F84.8 |
|  |  | 29981 | F84.5 |
|  |  |  | F84.8 |
|  |  | 29990 | F84.9 |
|  |  | 29991 | F84.9 |
|  |  | 30720 | F95.9 |
|  |  | 30721 | F95.0 |
|  |  | 30722 | F95.1 |
|  |  | 30723 | F95.2 |
|  |  | 3073 | F98.4 |
|  |  | 3076 | F98.0 |
|  |  | 3077 | F98.1 |
|  |  | 30921 | F93.0 |
|  |  | 31323 | F94.0 |
|  |  | 31389 | F93.8 |
|  |  |  | F94.1 |
|  |  |  | F98.8 |
|  |  | 3139 | F93.9 |
|  |  |  | F94.8 |
|  |  |  | F98.9 |
|  |  | 31230 | F63.9 |
|  |  | 31231 | F63.0 |
|  |  | 31232 | F63.2 |
|  |  | 31233 | F63.1 |
|  |  | 31234 | F63.81 |
|  |  | 31235 | F63.81 |
|  |  | 31239 | F63.3 |
|  |  |  | F63.89 |
|  |  | 29383 | F06.30 |
|  |  | 29600 | F30.10 |
|  |  | 29601 | F30.11 |
|  |  | 29602 | F30.12 |
|  |  | 29603 | F30.13 |
|  |  | 29604 | F30.2 |
|  |  | 29605 | F30.3 |
|  |  | 29606 | F30.4 |
|  |  | 29610 | F30.10 |
|  |  | 29611 | F30.11 |
|  |  | 29612 | F30.12 |
|  |  | 29613 | F30.13 |
|  |  | 29614 | F30.2 |
|  |  | 29615 | F30.3 |
|  |  | 29616 | F30.4 |
|  |  | 29620 | F32.9 |
|  |  | 29621 | F32.0 |
|  |  | 29622 | F32.1 |
|  |  | 29623 | F32.2 |
|  |  | 29624 | F32.3 |
|  |  | 29625 | F32.4 |
|  |  | 29626 | F32.5 |
|  |  | 29630 | F33.9 |
|  |  | 29631 | F33.0 |
|  |  | 29632 | F33.1 |
|  |  | 29633 | F33.2 |
|  |  | 29634 | F33.3 |
|  |  | 29635 | F33.41 |
|  |  | 29636 | F33.42 |
|  |  | 29640 | F31.10 |
|  |  | 29641 | F31.11 |
|  |  | 29642 | F31.12 |
|  |  | 29643 | F31.13 |
|  |  | 29644 | F31.2 |
|  |  | 29645 | F31.73 |
|  |  | 29646 | F31.74 |
|  |  | 29650 | F31.30 |
|  |  | 29651 | F31.31 |
|  |  | 29652 | F31.32 |
|  |  | 29653 | F31.4 |
|  |  | 29654 | F31.5 |
|  |  | 29655 | F31.75 |
|  |  | 29656 | F31.76 |
|  |  | 29660 | F31.60 |
|  |  | 29661 | F31.61 |
|  |  | 29662 | F31.62 |
|  |  | 29663 | F31.63 |
|  |  | 29664 | F31.64 |
|  |  | 29665 | F31.77 |
|  |  | 29666 | F31.78 |
|  |  | 2967 | F31.9 |
|  |  | 29680 | F31.9 |
|  |  | 29681 | F30.8 |
|  |  | 29682 | F32.89 |
|  |  | 29689 | F31.81 |
|  |  | 29690 | F39 |
|  |  | 29699 | F34.81 |
|  |  |  | F34.89 |
|  |  | 3004 | F34.1 |
|  |  | 311 | F32.9 |
|  |  | 3010 | F60.0 |
|  |  | 30110 | F34.0 |
|  |  | 30111 | F60.89 |
|  |  | 30112 | F34.1 |
|  |  | 30113 | F34.0 |
|  |  | 30120 | F60.1 |
|  |  | 30121 | F60.1 |
|  |  | 30122 | F21 |
|  |  | 3013 | F60.3 |
|  |  | 3014 | F60.5 |
|  |  | 30150 | F60.4 |
|  |  | 30151 | F68.12 |
|  |  | 30159 | F60.4 |
|  |  | 3016 | F60.7 |
|  |  | 3017 | F60.2 |
|  |  | 30181 | F60.81 |
|  |  | 30182 | F60.6 |
|  |  | 30183 | F60.3 |
|  |  | 30184 | F60.89 |
|  |  | 30189 | F60.89 |
|  |  | 3019 | F60.9 |
|  |  | 29381 | F06.2 |
|  |  | 29382 | F06.0 |
|  |  | 29500 | F20.89 |
|  |  | 29501 | F20.89 |
|  |  | 29502 | F20.89 |
|  |  | 29503 | F20.89 |
|  |  | 29504 | F20.89 |
|  |  | 29505 | F20.89 |
|  |  | 29510 | F20.1 |
|  |  | 29511 | F20.1 |
|  |  | 29512 | F20.1 |
|  |  | 29513 | F20.1 |
|  |  | 29514 | F20.1 |
|  |  | 29515 | F20.1 |
|  |  | 29520 | F20.2 |
|  |  | 29521 | F20.2 |
|  |  | 29522 | F20.2 |
|  |  | 29523 | F20.2 |
|  |  | 29524 | F20.2 |
|  |  | 29525 | F20.2 |
|  |  | 29530 | F20.0 |
|  |  | 29531 | F20.0 |
|  |  | 29532 | F20.0 |
|  |  | 29533 | F20.0 |
|  |  | 29534 | F20.0 |
|  |  | 29535 | F20.0 |
|  |  | 29540 | F20.81 |
|  |  | 29541 | F20.81 |
|  |  | 29542 | F20.81 |
|  |  | 29543 | F20.81 |
|  |  | 29544 | F20.81 |
|  |  | 29545 | F20.81 |
|  |  | 29550 | F20.89 |
|  |  | 29551 | F20.89 |
|  |  | 29552 | F20.89 |
|  |  | 29553 | F20.89 |
|  |  | 29554 | F20.89 |
|  |  | 29555 | F20.89 |
|  |  | 29560 | F20.5 |
|  |  | 29561 | F20.5 |
|  |  | 29562 | F20.5 |
|  |  | 29563 | F20.5 |
|  |  | 29564 | F20.5 |
|  |  | 29565 | F20.5 |
|  |  | 29570 | F25.9 |
|  |  | 29571 | F25.9 |
|  |  | 29572 | F25.9 |
|  |  | 29573 | F25.9 |
|  |  | 29574 | F25.9 |
|  |  | 29575 | F25.9 |
|  |  | 29580 | F20.89 |
|  |  | 29581 | F20.89 |
|  |  | 29582 | F20.89 |
|  |  | 29583 | F20.89 |
|  |  | 29584 | F20.89 |
|  |  | 29585 | F20.89 |
|  |  | 29590 | F20.9 |
|  |  | 29591 | F20.9 |
|  |  | 29592 | F20.9 |
|  |  | 29593 | F20.9 |
|  |  | 29594 | F20.9 |
|  |  | 29595 | F20.9 |
|  |  | 2970 | F22 |
|  |  | 2971 | F22 |
|  |  | 2972 | F22 |
|  |  | 2973 | F22 |
|  |  | 2978 | F22 |
|  |  | 2979 | F23 |
|  |  | 2980 | F32.3 |
|  |  |  | F33.3 |
|  |  | 2981 | F28 |
|  |  | 2982 | F44.89 |
|  |  | 2983 | F23 |
|  |  | 2984 | F23 |
|  |  | 2988 | F23 |
|  |  | 2989 | F29 |
|  |  | 2910 | F10.231 |
|  |  | 2911 | F10.96 |
|  |  | 2912 | F10.27 |
|  |  | 2913 | F10.951 |
|  |  | 2914 | F10.929 |
|  |  | 2915 | F10.950 |
|  |  | 2918 |  |
|  |  | 29181 | F10.239 |
|  |  | 29182 | F10.182 |
|  |  |  | F10.282 |
|  |  |  | F10.982 |
|  |  | 29189 | F10.159 |
|  |  |  | F10.180 |
|  |  |  | F10.181 |
|  |  |  | F10.188 |
|  |  |  | F10.259 |
|  |  |  | F10.280 |
|  |  |  | F10.281 |
|  |  |  | F10.288 |
|  |  |  | F10.959 |
|  |  |  | F10.980 |
|  |  | 2919 | F10.99 |
|  |  | 30300 | F10.229 |
|  |  | 30301 | F10.229 |
|  |  | 30302 | F10.229 |
|  |  | 30303 | F10.229 |
|  |  | 30390 | F10.20 |
|  |  | 30391 | F10.20 |
|  |  | 30392 | F10.20 |
|  |  | 30393 | F10.21 |
|  |  | 30500 | F10.10 |
|  |  | 30501 | F10.10 |
|  |  | 30502 | F10.10 |
|  |  | 30503 | F10.11 |
|  |  | 3575 | G62.1 |
|  |  | 4255 | I42.6 |
|  |  | 5353 |  |
|  |  | 53530 | K29.20 |
|  |  | 53531 | K29.21 |
|  |  | 5710 | K70.0 |
|  |  | 5711 | K70.10 |
|  |  | 5712 | K70.30 |
|  |  | 5713 | K70.9 |
|  |  | 76071 | P04.3 |
|  |  |  | Q86.0 |
|  |  | 9800 | T51.0X1A |
|  |  |  | T51.0X2A |
|  |  |  | T51.0X3A |
|  |  |  | T51.0X4A |
|  |  | 2920 | F19.939 |
|  |  | 29211 | F19.950 |
|  |  | 29212 | F19.951 |
|  |  | 2922 | F15.920 |
|  |  | 29281 | F19.921 |
|  |  | 29282 | F19.97 |
|  |  | 29283 | F19.96 |
|  |  | 29284 | F19.94 |
|  |  | 29285 | F11.182 |
|  |  |  | F11.282 |
|  |  |  | F11.982 |
|  |  |  | F13.182 |
|  |  |  | F13.282 |
|  |  |  | F13.982 |
|  |  |  | F14.182 |
|  |  |  | F14.282 |
|  |  |  | F14.982 |
|  |  |  | F15.182 |
|  |  |  | F15.282 |
|  |  |  | F15.982 |
|  |  |  | F19.182 |
|  |  |  | F19.282 |
|  |  |  | F19.982 |
|  |  | 29289 | F11.159 |
|  |  |  | F11.181 |
|  |  |  | F11.188 |
|  |  |  | F11.222 |
|  |  |  | F11.259 |
|  |  |  | F11.281 |
|  |  |  | F11.288 |
|  |  |  | F11.922 |
|  |  |  | F11.959 |
|  |  |  | F11.981 |
|  |  |  | F11.988 |
|  |  |  | F12.122 |
|  |  |  | F12.159 |
|  |  |  | F12.180 |
|  |  |  | F12.188 |
|  |  |  | F12.222 |
|  |  |  | F12.259 |
|  |  |  | F12.280 |
|  |  |  | F12.288 |
|  |  |  | F12.922 |
|  |  |  | F12.959 |
|  |  |  | F12.980 |
|  |  |  | F12.988 |
|  |  |  | F13.159 |
|  |  |  | F13.180 |
|  |  |  | F13.181 |
|  |  |  | F13.188 |
|  |  |  | F13.259 |
|  |  |  | F13.280 |
|  |  |  | F13.281 |
|  |  |  | F13.288 |
|  |  |  | F13.959 |
|  |  |  | F13.980 |
|  |  |  | F13.981 |
|  |  |  | F13.988 |
|  |  |  | F14.122 |
|  |  |  | F14.159 |
|  |  |  | F14.180 |
|  |  |  | F14.181 |
|  |  |  | F14.188 |
|  |  |  | F14.222 |
|  |  |  | F14.259 |
|  |  |  | F14.280 |
|  |  |  | F14.281 |
|  |  |  | F14.288 |
|  |  |  | F14.922 |
|  |  |  | F14.959 |
|  |  |  | F14.980 |
|  |  |  | F14.981 |
|  |  |  | F14.988 |
|  |  |  | F15.122 |
|  |  |  | F15.159 |
|  |  |  | F15.180 |
|  |  |  | F15.181 |
|  |  |  | F15.188 |
|  |  |  | F15.222 |
|  |  |  | F15.259 |
|  |  |  | F15.280 |
|  |  |  | F15.281 |
|  |  |  | F15.288 |
|  |  |  | F15.922 |
|  |  |  | F15.959 |
|  |  |  | F15.980 |
|  |  |  | F15.981 |
|  |  |  | F15.988 |
|  |  |  | F16.122 |
|  |  |  | F16.159 |
|  |  |  | F16.180 |
|  |  |  | F16.183 |
|  |  |  | F16.188 |
|  |  |  | F16.259 |
|  |  |  | F16.280 |
|  |  |  | F16.283 |
|  |  |  | F16.288 |
|  |  |  | F16.959 |
|  |  |  | F16.980 |
|  |  |  | F16.983 |
|  |  |  | F16.988 |
|  |  |  | F17.208 |
|  |  |  | F17.218 |
|  |  |  | F17.228 |
|  |  |  | F17.298 |
|  |  |  | F18.159 |
|  |  |  | F18.180 |
|  |  |  | F18.188 |
|  |  |  | F18.259 |
|  |  |  | F18.280 |
|  |  |  | F18.288 |
|  |  |  | F18.959 |
|  |  |  | F18.980 |
|  |  |  | F18.988 |
|  |  |  | F19.122 |
|  |  |  | F19.159 |
|  |  |  | F19.180 |
|  |  |  | F19.181 |
|  |  |  | F19.188 |
|  |  |  | F19.222 |
|  |  |  | F19.259 |
|  |  |  | F19.280 |
|  |  |  | F19.281 |
|  |  |  | F19.288 |
|  |  |  | F19.922 |
|  |  |  | F19.959 |
|  |  |  | F19.980 |
|  |  |  | F19.981 |
|  |  |  | F19.988 |
|  |  | 2929 | F19.99 |
|  |  | 30400 | F11.20 |
|  |  | 30401 | F11.20 |
|  |  | 30402 | F11.20 |
|  |  | 30403 | F11.21 |
|  |  | 30410 | F13.20 |
|  |  | 30411 | F13.20 |
|  |  | 30412 | F13.20 |
|  |  | 30413 | F13.21 |
|  |  | 30420 | F14.20 |
|  |  | 30421 | F14.20 |
|  |  | 30422 | F14.20 |
|  |  | 30423 | F14.21 |
|  |  | 30430 | F12.20 |
|  |  | 30431 | F12.20 |
|  |  | 30432 | F12.20 |
|  |  | 30433 | F12.21 |
|  |  | 30440 | F15.20 |
|  |  | 30441 | F15.20 |
|  |  | 30442 | F15.20 |
|  |  | 30443 | F15.21 |
|  |  | 30450 | F16.20 |
|  |  | 30451 | F16.20 |
|  |  | 30452 | F16.20 |
|  |  | 30453 | F16.21 |
|  |  | 30460 | F19.20 |
|  |  | 30461 | F19.20 |
|  |  | 30462 | F19.20 |
|  |  | 30463 | F19.21 |
|  |  | 30470 | F19.20 |
|  |  | 30471 | F19.20 |
|  |  | 30472 | F19.20 |
|  |  | 30473 | F19.21 |
|  |  | 30480 | F19.20 |
|  |  | 30481 | F19.20 |
|  |  | 30482 | F19.20 |
|  |  | 30483 | F19.21 |
|  |  | 30490 | F19.20 |
|  |  | 30491 | F19.20 |
|  |  | 30492 | F19.20 |
|  |  | 30493 | F19.21 |
|  |  | 30520 | F12.10 |
|  |  | 30521 | F12.10 |
|  |  |  | F12.90 |
|  |  | 30522 | F12.10 |
|  |  |  | F12.90 |
|  |  | 30523 | F12.11 |
|  |  | 30530 | F16.10 |
|  |  | 30531 | F16.10 |
|  |  | 30532 | F16.10 |
|  |  | 30533 | F16.11 |
|  |  | 30540 | F13.10 |
|  |  | 30541 | F13.10 |
|  |  | 30542 | F13.10 |
|  |  | 30543 | F13.11 |
|  |  | 30550 | F11.10 |
|  |  | 30551 | F11.10 |
|  |  | 30552 | F11.10 |
|  |  | 30553 | F11.11 |
|  |  | 30560 | F14.10 |
|  |  | 30561 | F14.10 |
|  |  | 30562 | F14.10 |
|  |  | 30563 | F14.11 |
|  |  | 30570 | F15.10 |
|  |  | 30571 | F15.10 |
|  |  | 30572 | F15.10 |
|  |  | 30573 | F15.11 |
|  |  | 30580 | F19.10 |
|  |  | 30581 | F19.10 |
|  |  | 30582 | F19.10 |
|  |  | 30583 | F19.11 |
|  |  | 30590 | F18.10 |
|  |  |  | F19.10 |
|  |  | 30591 | F18.10 |
|  |  |  | F19.10 |
|  |  | 30592 | F18.10 |
|  |  |  | F19.10 |
|  |  | 30593 | F18.11 |
|  |  |  | F19.11 |
|  |  | 64830 | O99.320 |
|  |  | 64831 | O99.321 |
|  |  |  | O99.322 |
|  |  |  | O99.323 |
|  |  |  | O99.324 |
|  |  | 64832 | O99.325 |
|  |  | 64833 | O99.321 |
|  |  |  | O99.322 |
|  |  |  | O99.323 |
|  |  | 64834 | O99.325 |
|  |  | 65550 | O35.5XX0 |
|  |  | 65551 | O35.5XX0 |
|  |  | 65553 | O35.5XX0 |
|  |  | 76072 | P04.49 |
|  |  | 76073 | P04.49 |
|  |  | 76075 | P04.41 |
|  |  | 7795 | P96.1 |
|  |  |  | P96.2 |
|  |  | 96500 | T40.0X1A |
|  |  |  | T40.0X2A |
|  |  |  | T40.0X3A |
|  |  |  | T40.0X4A |
|  |  | 96501 | T40.1X1A |
|  |  |  | T40.1X2A |
|  |  |  | T40.1X3A |
|  |  |  | T40.1X4A |
|  |  | 96502 | T40.3X1A |
|  |  |  | T40.3X2A |
|  |  |  | T40.3X3A |
|  |  |  | T40.3X4A |
|  |  | 96509 | T40.2X1A |
|  |  |  | T40.2X1A |
|  |  |  | T40.2X2A |
|  |  |  | T40.2X3A |
|  |  |  | T40.2X4A |
|  |  |  | T40.601A |
|  |  |  | T40.602A |
|  |  |  | T40.603A |
|  |  |  | T40.604A |
|  |  |  | T40.691A |
|  |  |  | T40.692A |
|  |  |  | T40.693A |
|  |  |  | T40.694A |
|  |  | V6542 | Z71.41 |
|  |  | E9500 |  |
|  |  | E9501 |  |
|  |  | E9502 |  |
|  |  | E9503 |  |
|  |  | E9504 |  |
|  |  | E9505 |  |
|  |  | E9506 |  |
|  |  | E9507 |  |
|  |  | E9508 |  |
|  |  | E9509 |  |
|  |  | E9510 |  |
|  |  | E9511 |  |
|  |  | E9518 |  |
|  |  | E9520 |  |
|  |  | E9521 |  |
|  |  | E9528 |  |
|  |  | E9529 |  |
|  |  | E9530 |  |
|  |  | E9531 |  |
|  |  | E9538 |  |
|  |  | E9539 |  |
|  |  | E954 | X71.8XXA |
|  |  |  | X71.9XXA |
|  |  | E9550 | X72.XXXA |
|  |  | E9551 | X73.0XXA |
|  |  | E9552 | X73.1XXA |
|  |  | E9553 | X73.2XXA |
|  |  | E9554 | X73.9XXA |
|  |  | E9555 | X75.XXXA |
|  |  | E9556 | X74.01XA |
|  |  | E9557 | X74.02XA |
|  |  | E9559 | X74.9XXA |
|  |  | E956 | X78.9XXA |
|  |  | E9570 | X80.XXXA |
|  |  | E9571 | Y92.009 |
|  |  | E9572 | X80.XXXA |
|  |  |  | Y92.828 |
|  |  |  | Y92.838 |
|  |  | E9579 | Y92.9 |
|  |  | E9580 | X81.8XXA |
|  |  | E9581 | X76.XXXA |
|  |  | E9582 | X77.2XXA |
|  |  | E9583 | X83.2XXA |
|  |  | E9584 | X83.1XXA |
|  |  | E9585 | X82.8XXA |
|  |  | E9586 | X83.0XXA |
|  |  | E9587 | X83.8XXA |
|  |  | E9588 | X83.8XXA |
|  |  | E9589 | X83.8XXA |
|  |  | E959 | X71.8XXS |
|  |  |  | X71.9XXS |
|  |  |  | X72.XXXS |
|  |  |  | X73.0XXA |
|  |  |  | X73.1XXA |
|  |  |  | X73.2XXA |
|  |  |  | X74.01XS |
|  |  |  | X74.02XS |
|  |  |  | X74.9XXS |
|  |  |  | X75.XXXS |
|  |  |  | X76.XXXS |
|  |  |  | X77.2XXS |
|  |  |  | X78.9XXS |
|  |  |  | X81.8XXS |
|  |  |  | X82.0XXS |
|  |  |  | X83.1XXS |
|  |  |  | X83.2XXS |
|  |  |  | X83.8XXS |
|  |  | V6284 | R45.851 |
|  |  | 3051 | F17.200 |
|  |  | 30510 |  |
|  |  | 30511 |  |
|  |  | 30512 |  |
|  |  | 30513 |  |
|  |  | 33392 | G21.0 |
|  |  | 7903 | R78.0 |
|  |  | V110 | Z65.8 |
|  |  | V111 | Z65.8 |
|  |  | V112 | Z65.8 |
|  |  | V113 | Z65.8 |
|  |  | V114 | Z86.51 |
|  |  | V118 | Z86.59 |
|  |  | V119 | Z86.59 |
|  |  | V154 |  |
|  |  | V1541 | Z91.410 |
|  |  | V1542 | Z91.411 |
|  |  |  | Z91.412 |
|  |  | V1549 | Z91.49 |
|  |  | V1582 | Z87.891 |
|  |  | V6285 | R45.850 |
|  |  | V663 | Z51.89 |
|  |  | V701 | Z04.6 |
|  |  | V702 | Z00.8 |
|  |  | V7101 | Z03.89 |
|  |  | V7102 | Z03.89 |
|  |  | V7109 | Z03.89 |
|  |  | V790 | Z13.89 |
|  |  | V791 | Z13.89 |
|  |  | V792 | Z13.4 |
|  |  | V793 | Z13.4 |
|  |  | V798 | Z13.4 |
|  |  | V799 | Z13.89 |
|  |  | 29389 | F06.1 |
|  |  |  | F53 |
|  |  | 2939 | F06.8 |
|  | Neurologic/CNS | 30011 | F44.4 |
|  |  |  | F44.6 |
|  |  | 30012 | F44.0 |
|  |  | 30013 | F44.1 |
|  |  | 30014 | F44.81 |
|  |  | 30015 | F44.91 |
|  |  | 30016 | F44.89 |
|  |  |  | F68.11 |
|  |  | 30019 | F68.8 |
|  |  | 3006 | F48.1 |
|  |  | 3007 | F45.21 |
|  |  |  | F45.22 |
|  |  | 30081 | F45.0 |
|  |  | 30082 | F45.1 |
|  |  |  | F45.9 |
|  |  | 3021 | F65.89 |
|  |  | 3022 | F65.4 |
|  |  | 3023 | F65.1 |
|  |  | 3024 | F65.2 |
|  |  | 30250 | F64.0 |
|  |  |  | Z87.890 |
|  |  | 30251 | F64.0 |
|  |  | 30252 | F64.0 |
|  |  | 30253 | F64.0 |
|  |  | 3026 | F64.2 |
|  |  | 30270 | R37 |
|  |  | 30271 | F52.0 |
|  |  | 30272 | F52.21 |
|  |  |  | F52.8 |
|  |  | 30273 | F52.31 |
|  |  | 30274 | F52.32 |
|  |  | 30275 | F52.4 |
|  |  | 30276 | F52.6 |
|  |  | 30279 | F52.1 |
|  |  |  | F52.8 |
|  |  | 30281 | F65.0 |
|  |  | 30282 | F65.3 |
|  |  | 30283 | F65.51 |
|  |  | 30284 | F65.52 |
|  |  | 30285 | F64.1 |
|  |  | 30289 | F65.81 |
|  |  |  | F65.89 |
|  |  |  | F66 |
|  |  | 3029 | F65.9 |
|  |  | 3060 | F45.8 |
|  |  | 3061 | F45.8 |
|  |  | 3062 | F45.8 |
|  |  | 3063 | F45.8 |
|  |  |  | F42.4 |
|  |  | 3064 | F45.8 |
|  |  | 30650 | F45.8 |
|  |  | 30651 | F52.5 |
|  |  | 30652 | F45.8 |
|  |  | 30653 | F45.8 |
|  |  | 30659 | F45.8 |
|  |  | 3066 | F45.8 |
|  |  | 3067 | F45.8 |
|  |  | 3068 | F45.8 |
|  |  |  | F59 |
|  |  | 3069 | F45.8 |
|  |  | 3071 | F50.00 |
|  |  | 30740 | F51.9 |
|  |  | 30741 | F51.02 |
|  |  |  | F51.09 |
|  |  | 30742 | F51.01 |
|  |  |  | F51.03 |
|  |  |  | F51.09 |
|  |  | 30743 | F51.19 |
|  |  | 30744 | F51.11 |
|  |  |  | F51.12 |
|  |  |  | F51.19 |
|  |  | 30745 | F51.8 |
|  |  | 30746 | F51.3 |
|  |  | 30747 | F51.8 |
|  |  | 30748 | F51.8 |
|  |  | 30749 | F51.8 |
|  |  | 30750 | F50.9 |
|  |  | 30751 | F50.2 |
|  |  | 30752 | F98.3 |
|  |  | 30753 | F98.21 |
|  |  | 30754 | F50.89 |
|  |  | 30759 | F50.81 |
|  |  |  | F50.82 |
|  |  |  | F50.89 |
|  |  |  | F98.29 |
|  |  | 30780 | F45.41 |
|  |  | 30781 | G44.209 |
|  |  | 30789 | F45.42 |
|  |  | 3101 | F07.0 |
|  |  | 316 | F54 |
|  |  | 64840 |  |
|  |  | 64841 | O99.340 |
|  |  | 64842 | O99.341 |
|  |  |  | O99.342 |
|  |  |  | O99.343 |
|  |  |  | O99.344 |
|  |  | 64843 | O99.341 |
|  |  |  | O99.342 |
|  |  |  | O99.343 |
|  |  | 64844 | O99.345 |
|  |  | V402 | F48.9 |
|  |  | V403 |  |
|  |  | V4031 | Z91.83 |
|  |  | V4039 | F69 |
|  |  | V409 | F69 |
|  |  | V673 | Z09 |
|  |  | 3320 | G20 |
|  |  | 340 | G35 |
|  |  | 3300 | E75.23 |
|  |  |  | E75.25 |
|  |  |  | E75.29 |
|  |  | 3301 | E75.02 |
|  |  |  | E75.19 |
|  |  |  | E75.4 |
|  |  | 3302 | G93.89 |
|  |  | 3303 | G93.9 |
|  |  | 3308 | F84.2 |
|  |  |  | G31.81 |
|  |  |  | G31.82 |
|  |  | 3309 | G31.9 |
|  |  | 3313 | G91.0 |
|  |  | 3314 | G91.1 |
|  |  | 3315 | G91.2 |
|  |  | 3316 | G31.85 |
|  |  | 3317 | G94 |
|  |  | 33181 | G93.7 |
|  |  | 33189 | G31.89 |
|  |  | 3319 | G31.9 |
|  |  | 3330 | G23.0 |
|  |  |  | G23.1 |
|  |  |  | G23.2 |
|  |  |  | G23.8 |
|  |  | 3331 | G25.0 |
|  |  |  | G25.1 |
|  |  |  | G25.2 |
|  |  | 3332 | G25.3 |
|  |  | 3333 | G25.61 |
|  |  |  | G25.69 |
|  |  | 3334 | G10 |
|  |  | 3335 | G25.4 |
|  |  |  | G25.5 |
|  |  | 3336 | G24.1 |
|  |  | 3337 |  |
|  |  | 33371 | G80.3 |
|  |  | 33372 | G24.02 |
|  |  | 33379 | G24.8 |
|  |  | 33381 | G24.5 |
|  |  | 33382 | G24.4 |
|  |  | 33383 | G24.3 |
|  |  | 33384 | G25.89 |
|  |  | 33385 | G24.01 |
|  |  | 33389 | G24.9 |
|  |  | 33390 | G25.9 |
|  |  | 33391 | G25.82 |
|  |  | 33393 | G25.83 |
|  |  | 33394 | G25.81 |
|  |  | 33399 | G25.89 |
|  |  |  | G25.9 |
|  |  | 3340 | G11.1 |
|  |  | 3341 | G11.4 |
|  |  | 3342 | G11.0 |
|  |  |  | G11.2 |
|  |  | 3343 | G11.1 |
|  |  | 3344 | G32.81 |
|  |  | 3348 | G11.3 |
|  |  |  | G11.8 |
|  |  | 3349 | G11.9 |
|  |  | 3350 | G12.0 |
|  |  | 33510 | G12.25 |
|  |  |  | G12.9 |
|  |  | 33511 | G12.1 |
|  |  | 33519 | G12.8 |
|  |  | 33520 | G12.21 |
|  |  | 33521 | G12.21 |
|  |  | 33522 | G12.22 |
|  |  | 33523 | G12.8 |
|  |  | 33524 | G12.23 |
|  |  | 33529 | G12.24 |
|  |  |  | G12.29 |
|  |  | 3358 | G12.8 |
|  |  | 3359 | G12.9 |
|  |  | 3360 | G95.0 |
|  |  | 3361 | G95.19 |
|  |  | 3362 | G32.0 |
|  |  | 3363 | G99.2 |
|  |  | 3368 | G95.89 |
|  |  | 3369 | G95.9 |
|  |  | 3370 |  |
|  |  | 33700 | G90.09 |
|  |  | 33701 | G90.01 |
|  |  | 33709 | G90.09 |
|  |  | 3371 | G99.0 |
|  |  | 3373 | G90.4 |
|  |  | 3379 | G90.9 |
|  |  | 3420 |  |
|  |  | 34200 | G81.00 |
|  |  | 34201 | G81.01 |
|  |  |  | G81.02 |
|  |  | 34202 | G81.03 |
|  |  |  | G81.04 |
|  |  | 3421 |  |
|  |  | 34210 | G81.10 |
|  |  | 34211 | G81.11 |
|  |  |  | G81.12 |
|  |  | 34212 | G81.13 |
|  |  |  | G81.14 |
|  |  | 34280 | G81.90 |
|  |  | 34281 | G81.91 |
|  |  |  | G81.92 |
|  |  | 34282 | G81.93 |
|  |  |  | G81.94 |
|  | Respiratory/ Pulmonary | 3429 |  |
|  |  | 34290 | G81.90 |
|  |  | 34291 | G81.91 |
|  |  |  | G81.92 |
|  |  | 34292 | G81.93 |
|  |  |  | G81.94 |
|  |  | 3430 | G80.1 |
|  |  | 3431 | G80.2 |
|  |  | 3432 | G80.0 |
|  |  | 3433 | G80.8 |
|  |  | 3434 | G80.2 |
|  |  | 3438 | G80.8 |
|  |  | 3439 | G80.9 |
|  |  | 3440 |  |
|  |  | 34400 | G82.50 |
|  |  | 34401 | G82.51 |
|  |  | 34402 | G82.52 |
|  |  | 34403 | G82.53 |
|  |  | 34404 | G82.54 |
|  |  | 34409 | G82.50 |
|  |  | 3441 | G82.20 |
|  |  | 3442 | G83.0 |
|  |  | 3443 |  |
|  |  | 34430 | G83.10 |
|  |  | 34431 | G83.11 |
|  |  |  | G83.12 |
|  |  | 34432 | G83.13 |
|  |  |  | G83.14 |
|  |  | 3444 |  |
|  |  | 34440 | G83.20 |
|  |  | 34441 | G83.21 |
|  |  |  | G83.22 |
|  |  | 34442 | G83.23 |
|  |  |  | G83.24 |
|  |  | 3445 | G83.30 |
|  |  | 34460 | G83.4 |
|  |  | 3448 |  |
|  |  | 34481 | G83.5 |
|  |  | 34489 | G83.81 |
|  |  |  | G83.84 |
|  |  |  | G83.89 |
|  |  | 3449 | G83.9 |
|  |  | 78072 | R53.2 |
|  |  | 7814 | R29.5 |
|  |  | 3450 |  |
|  |  | 34500 | G40.A01 |
|  |  |  | G40.A09 |
|  |  | 34501 | G40.A11 |
|  |  |  | G40.A19 |
|  |  | 3451 |  |
|  |  | 34510 | G40.309 |
|  |  |  | G40.401 |
|  |  |  | G40.409 |
|  |  | 34511 | G40.311 |
|  |  |  | G40.411 |
|  |  |  | G40.419 |
|  |  | 3452 | G40.A01 |
|  |  |  | G40.A09 |
|  |  |  | G40.A11 |
|  |  |  | G40.A19 |
|  |  | 3453 | G40.301 |
|  |  | 3454 |  |
|  |  | 34540 | G40.201 |
|  |  |  | G40.209 |
|  |  | 34541 | G40.211 |
|  |  |  | G40.219 |
|  |  | 3455 |  |
|  |  | 34550 | G40.101 |
|  |  |  | G40.109 |
|  |  | 34551 | G40.111 |
|  |  |  | G40.119 |
|  |  | 3456 |  |
|  |  | 34560 | G40.821 |
|  |  |  | G40.822 |
|  |  | 34561 | G40.823 |
|  |  |  | G40.824 |
|  |  | 3457 |  |
|  |  | 34570 | G40.101 |
|  |  |  | G40.109 |
|  |  | 34571 | G40.111 |
|  |  |  | G40.119 |
|  |  | 3458 |  |
|  |  | 34580 | G40.101 |
|  |  |  | G40.109 |
|  |  |  | G40.501 |
|  |  |  | G40.509 |
|  |  |  | G40.802 |
|  |  | 34581 | G40.111 |
|  |  |  | G40.119 |
|  |  |  | G40.804 |
|  |  | 3459 |  |
|  |  | 34590 | G40.901 |
|  |  |  | G40.909 |
|  |  | 34591 | G40.911 |
|  |  |  | G40.919 |
|  |  | 7803 |  |
|  |  | 78031 | R56.00 |
|  |  | 78032 | R56.01 |
|  |  | 78033 | R56.1 |
|  |  | 78039 | R56.9 |
|  |  | 3481 | G93.1 |
|  |  | 7800 |  |
|  |  | 78001 | R40.20 |
|  |  | 78003 | R40.3 |
|  |  | 78009 | R40.0 |
|  |  |  | R40.1 |
|  |  | 27700 | E84.9 |
|  |  | 27701 | E84.11 |
|  |  | 27702 | E84.0 |
|  |  | 27703 | E84.19 |
|  |  | 27709 | E84.8 |
|  |  | 4150 | I26.09 |
|  |  | 4151 |  |
|  |  | 41512 | !26.90 |
|  |  | 41513 | I26.92 |
|  |  | 41519 | I26.99 |
|  |  | 4160 | I27.0 |
|  |  | 4161 | I27.1 |
|  |  | 4162 | I27.82 |
|  |  | 4168 | !27.20 |
|  |  |  | !27.21 |
|  |  |  | !27.22 |
|  |  |  | !27.23 |
|  |  |  | !27.24 |
|  |  |  | I27.29 |
|  |  |  | I27.89 |
|  |  | 4169 | I27.81 |
|  |  |  | I27.9 |
|  |  | 4170 | I28.0 |
|  |  | 4171 | I28..1 |
|  |  | 4178 | I28.8 |
|  |  | 4179 | I28.9 |
|  |  | V1255 | Z86.711 |
|  |  | 490 | J40 |
|  |  | 4910 | J41.0 |
|  |  | 4911 | J41.1 |
|  |  | 4912 |  |
|  |  | 49120 | J44.9 |
|  |  | 49121 | J44.1 |
|  |  | 49122 | J44.0 |
|  |  | 4918 | J41.8 |
|  |  | 4919 | J42 |
|  |  | 4920 | J43.9 |
|  |  | 4928 | J43.9 |
|  |  | 494 |  |
|  |  | 4940 | J47.9 |
|  |  | 4941 | J47.1 |
|  |  | 496 | J44.9 |
|  |  | 49300 | J45.20 |
|  |  | 49301 | J45.22 |
|  |  | 49302 | J45.21 |
|  |  | 49310 | J45.20 |
|  |  | 49311 | J45.22 |
|  |  | 49312 | J45.21 |
|  |  | 49320 | J44.9 |
|  |  | 49321 | J44.0 |
|  |  | 49322 | J44.1 |
|  |  | 49381 | J45.990 |
|  |  | 49382 | J45.991 |
|  |  | 49390 | J45.909 |
|  |  |  | J45.998 |
|  |  | 49391 | J45.902 |
|  |  | 49392 | J45.901 |
|  |  | 5100 | J86.0 |
|  |  | 5109 | J86.9 |
|  |  | 5110 | J86.9 |
|  |  |  | J94.1 |
|  |  |  | J94.8 |
|  |  |  | J94.9 |
|  |  |  | R09.1 |
|  |  | 5111 | J90 |
|  |  |  | J94.2 |
|  |  | 5118 |  |
|  |  | 51189 | J90 |
|  |  |  | J94.2 |
|  |  |  | J94.8 |
|  |  | 5119 | J91.8 |
|  |  | 5120 | J93.0 |
|  |  | 5128 |  |
|  |  | 51281 | J93.11 |
|  |  | 51282 | J93.12 |
|  |  | 51283 | J93.81 |
|  |  | 51284 | J93.82 |
|  |  | 51289 | J93.83 |
|  |  |  | J93.9 |
|  |  | 5180 | J98.11 |
|  |  |  | J98.19 |
|  |  | 5181 | J98.2 |
|  |  | 5182 | J98.3 |
|  |  | 5173 | J99 |
|  |  | 5185 |  |
|  |  | 51851 | J95.821 |
|  |  |  | J96.00 |
|  |  | 51852 | J95.1 |
|  |  |  | J95.2 |
|  |  |  | J95.3 |
|  |  | 51853 | J95.822 |
|  |  |  | J96.20 |
|  |  | 51881 | J96.00 |
|  |  |  | J96.90 |
|  |  | 51882 | J80 |
|  |  |  | R06.03 |
|  |  | 51883 | J96.10 |
|  |  | 51884 | J96.20 |
|  |  | 7991 | R09.2 |
|  |  | V461 | Z53.09 |
|  |  | V4611 | Z99.11 |
|  |  | V4612 | Z99.12 |
|  |  | V4613 | Z99.11 |
|  |  | V4614 | J95.850 |
|  |  | V462 | Z99.81 |
|  |  | 4950 | J67.0 |
|  |  | 4951 | J67.1 |
|  |  | 4952 | J67.2 |
|  |  | 4953 | J67.3 |
|  |  | 4954 | J67.4 |
|  |  | 4955 | J67.5 |
|  |  | 4956 | J67.6 |
|  |  | 4957 | J67.7 |
|  |  | 4958 | J67.8 |
|  |  | 4959 | J67.9 |
|  |  | 500 | J60 |
|  |  | 501 | J61 |
|  |  | 502 | J62.8 |
|  |  | 503 | J63.0 |
|  |  |  | J63.1 |
|  |  |  | J63.2 |
|  |  |  | J63.3 |
|  |  |  | J63.4 |
|  |  |  | J63.5 |
|  |  |  | J63.6 |
|  |  | 504 | J66.0 |
|  |  |  | J66.1 |
|  |  |  | J66.2 |
|  |  |  | J66.8 |
|  |  | 505 | J64 |
|  |  | 5060 | J68.0 |
|  |  | 5061 | J68.1 |
|  |  | 5062 | J68.2 |
|  |  | 5063 | J68.3 |
|  |  | 5064 | J68.4 |
|  |  | 5069 | J68.9 |
|  |  | 5071 | J69.1 |
|  |  | 5078 | J69.8 |
|  |  | 5080 | J70.0 |
|  |  | 5081 | J70.1 |
|  |  | 5082 | J70.5 |
|  |  | 5088 | J70.8 |
|  |  | 5089 | J70.9 |
|  |  | 5131 | J85.3 |
|  |  | 514 | J18.2 |
|  |  |  | J18.1 |
|  |  | 515 | J84.10 |
|  |  |  | J84.89 |
|  |  | 5160 | J84.01 |
|  |  | 5161 | J84.03 |
|  |  | 5162 | J84.02 |
|  |  | 5163 |  |
|  |  | 51630 | J84.111 |
|  |  | 51631 | J84.113 |
|  |  | 51632 | J84.113 |
|  |  | 51633 | J84.114 |
|  |  | 51634 | J84.115 |
|  |  | 51635 | J84.2 |
|  |  | 51636 | J84.116 |
|  |  | 51637 | J84.117 |
|  |  | 5164 | J84.81 |
|  |  | 5165 | J84.82 |
|  |  | 51661 | J84.841 |
|  |  | 51662 | J84.842 |
|  |  | 51663 | J84.843 |
|  |  | 51664 | J84.843 |
|  |  | 51669 | J84.848 |
|  |  | 5168 | J84.09 |
|  |  | 5169 | J84.9 |
|  |  | 5172 | M34.81 |
|  |  | 5178 | J99 |
|  |  | 5183 | J82 |
|  |  | 5184 | J81.0 |
|  |  | 51889 | J98.4 |
|  |  | 5194 | J98.6 |
|  |  | 5198 | J98.8 |
|  |  | 5199 | J98.9 |
|  |  | 7825 | R23.0 |
|  |  | 78600 | R06.9 |
|  |  | 78601 | R06.4 |
|  |  | 78602 | R06.01 |
|  |  | 78603 | R06.81 |
|  |  | 78604 | R06.3 |
|  |  | 78605 | R06.02 |
|  |  | 78606 | R06.82 |
|  |  | 78607 | R06.2 |
|  |  | 78609 | R06.00 |
|  |  |  | R06.09 |
|  |  |  | R06.3 |
|  |  |  | R06.83 |
|  |  |  | R06.89 |
|  |  | 7862 | R05 |
|  |  | 7863 |  |
|  |  | 78630 | R04.2 |
|  |  |  | R04.9 |
|  |  | 78631 | R04.81 |
|  |  | 78639 | R04.89 |
|  |  | 7864 | R09.3 |
|  |  | 78652 | R07.1 |
|  |  |  | R07.81 |
|  |  | 7866 | R22.2 |
|  |  | 7867 | R09.89 |
|  |  | 7868 | R06.6 |
|  |  | 7869 | R06.89 |
|  |  | 7931 |  |
|  |  | 79311 | R91.1 |
|  |  | 79319 | R91.8 |
|  |  | 7942 | R94.2 |
|  |  | V126 |  |
|  |  | V1260 | Z87.09 |
|  |  | V1261 | Z87.01 |
|  |  | V1269 | Z87.09 |
|  |  | V426 | Z94.2 |
|  |  | 769 | F22.0 |
