# Supplementary material for: Racial Disparities in Outcomes of Delivery and Cardiac Complications Among Pregnant Women with Congenital Heart Disease
Source: J Racial Ethn Health Disparities. 2024 Feb 28;12(2):1159–69. doi: 10.1007/s40615-024-01950-0 (PMC11913936; doi:10.1007/s40615-024-01950-0)
Supplement: Supplementary file 2 — Supplementary file2 (DOCX 18 KB) [file 40615_2024_1950_MOESM2_ESM.docx]

**Supplemental Table S2.** Fetal events stratified by race and income quartile

|  | Unadjusted | | |  | Adjusted | |
| --- | --- | --- | --- | --- | --- | --- |
|  | Rates | OR (95% CI) | *p* |  | OR (95% CI) | *p* |
| Income Quartile - I |  |  |  |  |  |  |
| Black vs. White | 34.0 vs. 31.5 | 1.1 (0.9-1.4) | 0.341 |  | 1.1 (0.8-1.4) | 0.565 |
| Black vs. Hispanic | 34.0 vs. 29.0 | 1.3 (0.9-1.6) | 0.095 |  | 1.2 (0.9-1.6) | 0.170 |
| Black vs. Other | 34.0 vs. 32.1 | 1.1 (0.7-1.6) | 0.683 |  | 1.0 (0.7-1.6) | 0.878 |
| White vs. Hispanic | 31.5 vs. 29.0 | 1.1 (0.9-1.4) | 0.310 |  | 1.1 (0.9-1.4) | 0.296 |
| White vs. Other | 31.5 vs. 32.1 | 0.9 (0.7-1.4) | 0.889 |  | 1.0 (0.7-1.4) | 0.851 |
| Hispanic vs. Other | 29.0 vs. 32.1 | 0.9 (0.6-1.3) | 0.488 |  | 0.9 (0.6-1.3) | 0.450 |
| Income Quartile - II |  |  |  |  |  |  |
| Black vs. White | 35.3 vs. 29.2 | 1.3 (0.9-1.8) | 0.074 |  | 1.3 (0.9-1.7) | 0.131 |
| Black vs. Hispanic | 35.3 vs. 26.0 | 1.6 (1.1-2.2) | 0.018 |  | 1.5 (1.1-2.2) | 0.031 |
| Black vs. Other | 35.3 vs. 42.4 | 0.7 (0.5-1.2) | 0.193 |  | 0.7 (0.5-1.1) | 0.146 |
| White vs. Hispanic | 29.2 vs. 26.0 | 1.2 (0.9-1.5) | 0.217 |  | 1.2 (0.9-1.5) | 0.202 |
| White vs. Other | 29.2 vs. 42.4 | 0.6 (0.4-0.8) | 0.002 |  | 0.6 (0.4-0.8) | 0.003 |
| Hispanic vs. Other | 26.0 vs. 42.4 | 0.25 (0.3-0.7) | <.001 |  | 0.5 (0.3-0.7) | <.001 |
| Income Quartile - III |  |  |  |  |  |  |
| Black vs. White | 34.2 vs. 26.8 | 1.4 (1.1-2.0) | 0.030 |  | 1.4 (0.9-1.9) | 0.059 |
| Black vs. Hispanic | 34.2 vs. 34.2 | 1.0 (0.7-1.5) | 0.999 |  | 1.0 (0.7-1.4) | 0.880 |
| Black vs. Other | 34.2 vs. 31.2 | 1.2 (0.7-1.8) | 0.537 |  | 1.1 (0.7-1.8) | 0.625 |
| White vs. Hispanic | 26.8 vs. 34.2 | 0.7 (0.5-0.9) | 0.007 |  | 0.7 (0.5-0.9) | 0.009 |
| White vs. Other | 26.8 vs. 31.2 | 0.8 (0.6-1.1) | 0.213 |  | 0.8 (0.6-1.2) | 0.247 |
| Hispanic vs. Other | 34.2 vs. 31.2 | 1.1 (0.8-1.7) | 0.490 |  | 1.2 (0.8-1.7) | 0.480 |
| Income Quartile - IV |  |  |  |  |  |  |
| Black vs. White | 45.3 vs. 26.2 | 2.3 (1.6-3.4) | <.001 |  | 2.3 (1.5-3.4) | <.001 |
| Black vs. Hispanic | 45.3 vs. 30.8 | 1.9 (1.1-3.0) | 0.013 |  | 1.8 (1.1-3.0) | 0.017 |
| Black vs. Other | 45.3 vs. 29.9 | 1.9 (1.2-3.1) | 0.005 |  | 1.9 (1.2-3.0) | 0.008 |
| White vs. Hispanic | 26.2 vs. 30.8 | 0.8 (0.6-1.1) | 0.168 |  | 0.8 (0.6-1.1) | 0.196 |
| White vs. Other | 26.2 vs. 29.9 | 0.8 (0.6-1.1) | 0.233 |  | 0.8 (0.6-1.1) | 0.229 |
| Hispanic vs. Other | 30.8 vs. 29.9 | 1.0 (0.7-1.6) | 0.832 |  | 1.0 (0.7-1.5) | 0.883 |
